# A SRC-slug-TGFβ2 signaling axis drives poor outcomes in triple-negative breast cancers

**DOI:** 10.1186/s12964-024-01793-6

**Published:** 2024-09-26

**Authors:** Charlotte Zoe Angel, Shannon Beattie, Ezanee Azlina Mohamad Hanif, Micheal P. Ryan, Francisco D. C. Guerra Liberal, Shu-Dong Zhang, Scott Monteith, Niamh E. Buckley, Emma Parker, Shannon Haynes, Alexander J. McIntyre, Paula Haddock, Madina Sharifova, Cristina M. Branco, Paul B. Mullan

**Affiliations:** 1 Patrick G Johnston Centre for Cancer Research, Queen’s University Belfast, Belfast, Northern Ireland; 2 C-TRIC Building, Altnagelvin Area Hospital, Ulster University, Derry, Northern Ireland; 3 School of Pharmacy, Queen’s University Belfast, Belfast, Northern Ireland

## Abstract

**Background:**

Treatment options for the Triple-Negative Breast Cancer (TNBC) subtype remain limited and the outcome for patients with advanced TNBC is very poor. The standard of care is chemotherapy, but approximately 50% of tumors develop resistance.

**Methods:**

We performed gene expression profiling of 58 TNBC tumor samples by microarray, comparing chemosensitive with chemoresistant tumors, which revealed that one of the top upregulated genes was *TGFβ2.* A connectivity mapping bioinformatics analysis predicted that the SRC inhibitor Dasatinib was a potential pharmacological inhibitor of chemoresistant TNBCs. Claudin-low TNBC cell lines were selected to represent poor-outcome, chemoresistant TNBC, for in vitro experiments and in vivo models.

**Results:**

In vitro, we identified a signaling axis linking SRC, AKT and ERK2, which in turn upregulated the stability of the transcription factors, Slug and Snail. Slug was shown to repress *TGFβ2-antisense 1* to promote *TGFβ2* signaling, upregulating cell survival *via* apoptosis and DNA-damage responses. Additionally, an orthotopic allograft in vivo model demonstrated that the SRC inhibitor Dasatinib reduced tumor growth as a single agent, and enhanced responses to the TNBC mainstay drug, Epirubicin.

**Conclusion:**

Targeting the SRC-Slug-TGFβ2 axis may therefore lead to better treatment options and improve patient outcomes in this highly aggressive subpopulation of TNBCs.

**Supplementary Information:**

The online version contains supplementary material available at 10.1186/s12964-024-01793-6.

## Abstract (plain language summary)

In our study, we focused on a particular subtype of aggressive breast cancer called Triple-Negative Breast Cancer (TNBC). We investigated a complex series of events that contribute to poor outcomes in this disease and uncovered a crucial signaling cascade driving tumor growth and progression.

At the core of this signaling cascade are three key proteins: SRC, AKT, and ERK2. Together, they form a pathway that activates a transcription factor called Slug. Transcription factors act like molecular switches, controlling the expression of genes. Once Slug is activated, it strongly suppresses genes that would normally restrict cell growth and cell spread.

One of the genes downregulated by Slug is TGFB2-AS1. This product of the TGFB2-AS1 gene normally controls levels of its target protein called TGF-beta2 (TGFB2), a protein which has roles in cell growth, cell migration and differentiation. Slug downregulation of TGFB2-AS1 results in higher TGFB2 levels, and this in turn contributes to the uncontrolled growth and spread of cancer cells. TGFB2, and other proteins in this pathway (SRC, AKT, ERK2, and a Slug interactor called LSD1) all maintain the stability of Slug, meaning that Slug levels remain high and drive the aggressive features of this subtype of breast cancer.

Overall, our research sheds light on the intricate molecular mechanisms driving aggressive TNBC. It also identifies potential targets for future therapies, aimed at disrupting this harmful signaling pathway and potentially improving patient outcomes for this disease.

**Supplementary Information:**

The online version contains supplementary material available at 10.1186/s12964-024-01793-6.

## Introduction

Breast cancer is the leading cause of cancer-related death in women [40], and is a heterogeneous disease of molecular subgroups (Luminal A, Luminal B, HER2 overexpressing, Basal-like/Triple-Negative (TNBC)), each with distinct biologies and clinical outcomes [27, 37]. TNBC accounts for approximately 15% of all breast cancer patients [45]. TNBC lacks oestrogen and progesterone hormone receptors (ERα and PR), and does not display amplification of the HER2 growth factor receptor, making TNBCs refractory to therapies targeting those receptors (such as Tamoxifen and Trastuzumab). Around 20% of TNBCs have mutations in the DNA-damage repair protein BRCA1 [10], meaning that these tumors respond to platinum agents and PARP inhibitors, such as Olaparib [45]. The standard treatment for TNBC is surgery and chemotherapy, such as an anthracycline plus a taxane, an antimetabolite, or a DNA alkylating agent. In the UK, patients are typically prescribed 5-Fluorouracil, Epirubicin and Cyclophosphamide (the ‘FEC’ cocktail) which is curative in less than half of patients [22, 28]. This poor prognosis reflects an intrinsic aggressive biology, with TNBC being more likely to progress rapidly, to metastasize to visceral organs, with limited treatment options and poorer outcomes than the other subgroups [9, 44]. There is therefore an urgent need to develop additional therapeutics, in particular for the ‘poor-outcome’ subpopulations with the TNBC subgroup.

To demonstrate subpopulations within the TNBC subgroup, TNBCs have been previously stratified by the Lehmann-4 system (Mesenchymal, Luminal Androgen Receptor positive, Basal-like 1 and Basal-like 2) [8], 17]. Additionally, 25–39% of TNBC tumors are classed as ‘claudin-low’ TNBCs, a subpopulation characterized as being chemoresistant, often metastatic and associated with poor patient outcomes [30], but the molecular mechanisms remain poorly understood. Clearly, improving the treatment responses in this subpopulation of TNBCs is a priority for better clinical management of chemoresistant TNBCs.

In this study, we analyzed 58 patient tumor samples and observed that expression of the cytokine *TGFβ2* was associated with poor responses to FEC. Anti-TGFβ therapies have been explored extensively in cancer, with some promise in in vitro and preclinical models, but clinical trials have been disappointing [41]. We propose that new approaches to specifically reduce *TGFβ2* expression in tumors or to inhibit its associated biology, could improve treatment responses for poor outcome TNBCs. We show for the first time that *TGFβ2* expression is regulated by a SRC driven mechanism, *via* AKT, ERK2, LSD1 and the Slug and Snail transcription factors. We conclude that this SRC-Slug-TGFβ2 axis may contribute to chemotherapy resistance in an aggressive subpopulation of TNBC tumors, and targeting this pathway may help to improve outcomes for this aggressive breast cancer subtype.

## Methods

### Microarray analysis of ex vivo TNBC

Tumour samples from 78 patients were collected from the Northern Ireland Biobank, following ethical and research governance approval. All patients had been diagnosed with TNBC and treated with chemotherapy at the Northern Ireland Cancer Centre, according to the standard of care procedures in the UK. Tumour samples were stored in FFPE blocks and total RNA was isolated using the RNeasy FFPE Kit (Qiagen, Hilden, Germany), and converted to cDNA using the Transcriptor First Strand cDNA synthesis kit (Roche, Basel, Switzerland). Microarray profiling was performed using an Xcel array (Almac, Craigavon, Northern Ireland) with 58 samples deemed suitable for analysis. Clinical data points for patients in this study can be found in Supplementary Table 1. Following profiling, data were analyzed on a Partek Genomic Suite v6. The clinical discriminators were: (i) no relapse or any other adverse events occurring within 3 years following chemotherapy (‘good-outcome’); and (ii) relapse within 3 years (‘poor-outcome’). Given the highly heterogeneous nature of the profiles, all samples were analyzed with fold-changes > 1.5 (upregulation) and < 1.5 (downregulation) at significance value *p* < 0.05 without FDR correction.

### Bioinformatics

Selected gene targets were used as query signatures to search a connectivity mapping database, a subset of LINCS drug-induced gene expression profiles based on FDA approved drugs with ~ 114k instances of drug treatments covering ~ 1400 drugs, which is an expanded collection of the QUADrATiC reference gene expression database. The data processing flow from LINCS data to create the QUADrATiC database was described in [26]. Pathway analysis was performed using ENRICHR [2].

### Cell culture and maintenance

Cell lines were obtained from ATCC and cultured as described [23]. Hs578T, MDA-MB-231, MDA-MB-468, MDA-MB-436, MCF7, and Phoenix AMPHO were cultured in DMEM medium with 10% foetal bovine serum (FBS) (Gibco, Billings, USA), HCC-3153 and HCC-1806 in RPMI 1640 medium with 10% FBS, HME1 in HUMEC medium, Bovine Pituitary Growth Serum (25 mg) (Gibco, Billings, USA).

### Gene expression modification

Cells were transfected with siRNA (Eurofins, Luxembourg City, Luxembourg) at a final concentration of 20nM using Lipofectamine^®^ RNAimax (Thermo Fisher Scientific, Waltham, USA) and analyzed relative to GFP-targeting scrambled control (siSCR), as described [23]. Overexpression transfections were performed by subcloning into the pBabePuro vector, transfected alongside retrovirus packaging plasmid VSVG into Phoenix AMPHO cells with GeneJuice (Merck, Darmstadt, Germany), followed by infection of target cell lines using 0.45 μm-filtered viral media with 8 µg/ml Polybrene (Sigma Aldrich, Burlington, USA) for 24 h and then positive clones were selected using 1 µg/ml Puromycin (Sigma Aldrich, Burlington, USA). Drugs are listed in Supplementary Material Appendix 1.

### Phenotypic assays

For colony forming assay, cells were seeded at low density (50–100 cells/cm^2^), treated after 24 h and then incubated as described [13]. For migration assay, a scratch was created through a confluent monolayer by sterile pipette tip and analyzed for up to 16 h by light microscopy, with data analysis using an ImageJ plugin [38].

### MTT and cell drug treatments

Cells were seeded at 4,000 cells per well of a 96-well plate with 6 technical replicates, stained with MTT (Sigma Aldrich, Burlington, USA) for 1–2 h according to the manufacturer’s instructions, then the media was aspirated and MTT dye extracted with 100% DMSO (Sigma Aldrich, Burlington, USA) on a rocking platform for 10 min, with absorbances (OD = 590 nm) measured by FLUOstar Omega microplate reader (BMG Labtech, Baden-Wuerttemberg, Germany). For cell viability assays, a 2 h timepoint was used as the reference value, and compared with the 72 h timepoint. For drugging studies, the media was replaced after 24 h with titrations of drug-containing media adjusted for DMSO content, or a DMSO-only control, with MTT performed 72 h later.

### Real-time quantitative PCR (RT-qPCR)

Total RNA was isolated using TriPure reagent (Sigma Aldrich, Burlington, USA) and converted to cDNA using the Transcriptor First Strand cDNA synthesis kit (Roche, Basel, Switzerland), and real-time quantitative PCR (RT-qPCR) was performed as described [23]. Expression was normalized to the mean levels of housekeeping genes SHDA and HPRT (for claudin-low cells) or the mean of GAPDH and ACTIN (all other cell lines). Expression of pre-miR-205 was normalized to pre-RNU6. Primer sequences are available in Appendix 2.

### Western blot

Cell or tissue pellets were lysed in ice-cold RIPA buffer (50mM Tris pH7.5, 150mM NaCl, 1% Igepal, 0.5% Sodium Deoxycholate, 0.1% SDS) supplemented with cOmplete Mini protease inhibitor cocktail and PhosSTOP phosphatase inhibitor cocktail (Merck, Darmstadt, Germany). Lysates were separated by SDS polyacrylamide gel and transferred to PVDF membrane followed by immunoblotting. Antibodies are listed in Appendix 3.

### DNA damage response analysis

Cells were seeded onto cover slides and treated with Dasatinib at the IC50 for 48 h before irradiation. X-ray irradiations, staining and immunofluorescence were performed as described [13].

### In vivo experiments

In vivo experiments were conducted under project license PPL2859, approved by the NI Department of Health/United Kingdom Home Office and the institutional ethical committee, and compliant with the ethical principles of the Animals (Scientific Procedures) Act 1986 and reported in compliance with the ARRIVE guidelines, and described in full in Supplementary Methods. No animals were excluded from the study or analysis.

### Supplementary data methods

Methods pertaining to Supplementary Data are described in Supplementary Methods 2.

### Statistics

All statistics were carried out using GraphPad Prism v7 where p is considered significant at *<0.05, **<0.005, ***<0.001. All experiments represent three independent measurements, Supplementary Figures represent a minimum of two independent measurements.

## Results

### TGFβ2 is a marker and a driver of poor-outcome TNBC

We applied a principal component analysis to the microarray data, comparing tumors that responded to FEC chemotherapy (‘good outcome’) with those that did not (‘poor outcome’). Of note, TGFβ2 was one of the most highly upregulated genes in the ‘poor-outcome’ cohort, increased up to LOG2 2.65-fold, across multiple probesets (Supplementary Table 2). This aligned with the established oncogenic role of *TGFβ2* [41], making TGFβ2 a gene of particular interest for our studies. To elucidate the wider signaling network of TGFβ2 in the context of poor-outcome TNBCs, we used a connectivity mapping analysis (CMA) to search for small molecule compounds capable of regulating TGFβ2 mRNA expression in the claudin-low cell line, MDA-MB-231 [39]. This CMA highlighted that the SRC inhibitor Dasatinib was a potential regulator of the chemoresistant gene expression signature (Supplementary Fig. 1A).

**Fig. 1:**
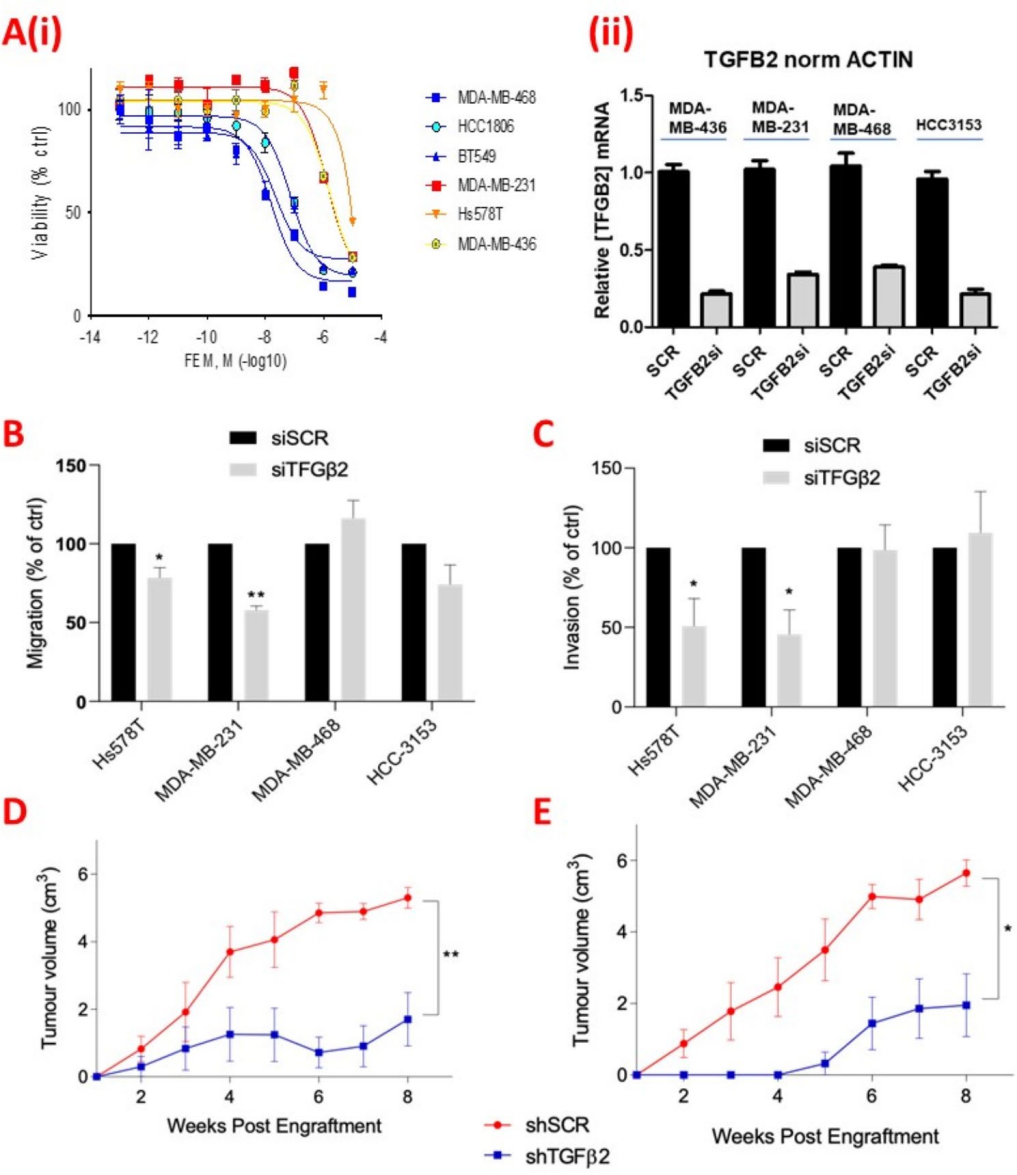
TGFβ2 is upregulated in poor-outcome TNBC. (**A**) (i) Line graph showing dose responses (as measured by an MTT viability assay) of claudin-low cell lines (MDA-MB-231, Hs578T and MDA-MB-436) and other TNBC subtypes (HCC1806, BT20, HCC70) to a FEM chemotherapy cocktail. (ii) Bar graph showing TGFβ2 qPCR values following knockdown of TGFβ2 by siRNA (TGFβ2si), relative to scrambled control siRNA (SCR) in claudin-low cell lines (Hs578T and MDA-MB-231) and basal-like cell lines (MDA-MB-468 and HCC-3153). Beta-Actin mRNA was used for normalisation, with TGFβ2si values expressed as a fraction of SCR control. (**B**) Bar graph demonstrating cell migration rates (measured using a ‘wound scratch assay’) following knockdown of TGFβ2 by siRNA (siTGFβ2), relative to scrambled control siRNA (siSCR) in claudin-low cell lines (Hs578T and MDA-MB-231) and basal-like cell lines (MDA-MB-468 and HCC-3153). Graph depicts scratch coverage by siTGFβ2-treated cells as a percentage of siSCR-treated cells, 72 h post-transfection (mean + SD of three independent experiments, analyzed by t-test, where *p < 0.05, **p < 0.005, ***p < 0.001). (**C**) Bar graph demonstrating cell invasion through matrigel of claudin-low (Hs578T and MDA-MB-231) and basal-like (MDA-MB-468 and HCC-3153) TNBC cell lines treated with siTGFβ2 relative to siSCR. Graph depicts the rates which crystal violet stained siTGFβ2-treated cells invade across a matrigel layer, as a percentage relative to siSCR-treated cells, 72 h post-transfection (mean + SD of three independent experiments, analyzed by t-test, where *p < 0.05, **p < 0.005, ***p < 0.001). (**D**) Line graph demonstrating mean tumor volume of in vivo xenograft TNBC tumors derived from MDA-MB-231 cells with constitutive expression of shRNA against TGFβ2 (shTGFβ2), compared with scrambled control shRNA (shSCR) (*n* = 6 per group, mean + SD of tumor volume, analyzed by t-test, where *p < 0.05, **p < 0.005, ***p < 0.001). (**E**) Line graph demonstrating mean tumor volume of in vivo xenograft TNBC tumors derived from Hs578T cells with constitutive expression of shRNA against TGFβ2 (shTGFβ2) compared with scrambled control shRNA (shSCR) (*n* = 6 per group, mean + SD of tumor volume, analyzed by t-test, where *p < 0.05, **p < 0.005, ***p < 0.001)

We next screened TNBC cell lines to identify which lines best represented poor outcome TNBC, to further explore the role of TGFβ2, and to test chemoresistance. We assessed breast cancer cell lines’ innate resistance to chemotherapy by determining IC50 values for the chemotherapy cocktail ‘FEM’ (5-Fluorouracil, Epirubicin and Mitomycin C, with Mitomycin C the in vitro substitute for Cyclophosphamide). As predicted, the claudin-low cell lines investigated (MDA-MB-231, Hs578T and MDA-MB-436) displayed the highest FEM chemoresistance, compared with basal-like cell lines, MDA-MB-468, HCC-1806 and BT549 (Fig. 1A(i)).

To determine if TGFβ2 signaling contributed to aggressive behaviour, we used siRNA to knockdown *TGFβ2* expression in claudin-low and basal-like cell lines (knockdowns shown in Fig. 1A(ii)). Equivalent TGFβ2 knockdowns significantly inhibited cell migration and invasion in the 2 claudin-low cell lines in particular, with no obvious similar phenotypes in the 2 basal-like cell lines (Fig. 1B-C). We also generated MDA-MB-231 and Hs578T cell lines with stable *TGFβ2* knockdown by shRNA (shTGFβ2) and established subcutaneous tumors in SCID mice. *TGFβ2* shRNA knockdown tumors displayed reduced growth compared with scrambled control shRNA (Fig. 1D-E), corroborating that TGFβ2 is also important for TNBC tumor development and aggressiveness in vivo.

### SRC maintains EMT signaling in TNBC

**Fig. 2:**
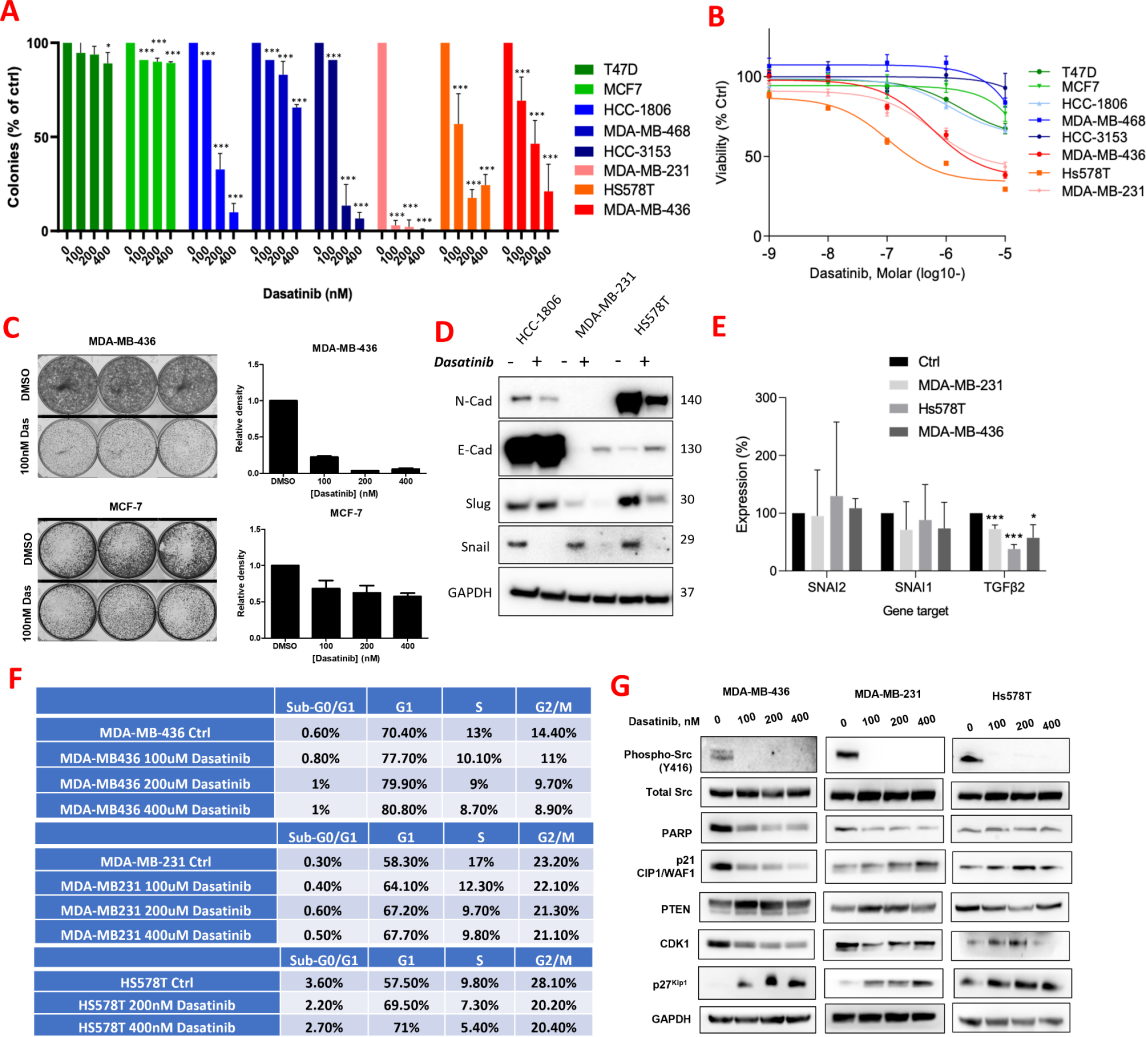
The SRC inhibitor Dasatinib is a potential drug treatment for poor-outcome TNBC. (**A**) Bar graph demonstrating the number of crystal violet stained colonies observed in clonogenic assays, with multiple doses of Dasatinib (x-axis) in TNBC cell lines (HCC-1806, MDA-MB-468, HCC-3153, MDA-MB-231, Hs578T, MDA-MB-436) and hormone receptor positive cell lines (MCF7 and T47D), with clonogenicities expressed as a percentage of control treatment (DMSO). Graph depicts mean + SD of three independent experiments, analyzed by t-test, where *p < 0.05, **p < 0.005, ***p < 0.001. **B** Line graph depicting the dose response analysis of Dasatinib (as measured by MTT viability assay) in TNBC cell lines (HCC-1806, MDA-MB-468, HCC-3153, MDA-MB-231, Hs578T, MDA-MB-436) and hormone receptor positive cell lines (MCF7 and T47D), relative to the control treatment (DMSO). (**C**) Clonogenic assays showing the relative sensitivities to Dasatinib of a claudin-low cell line (MDA-MB-436) compared to a luminal breast cancer cell line (MCF7). Cells were seeded at low density and then exposed to the indicated concentrations of Dasatinib for up to 7 days. (**D**) Western blot analysis of the levels of EMT-associated markers following Dasatinib treatment (at the IC50 for 72 h) in TNBC cell lines HCC-1806, MDA-MB-231 and Hs578T. Membranes were immunoblotted for the epithelial marker E-cadherin (E-cad), and the mesenchymal markers N-cadherin (N-cad), Slug and Snail, with levels measured compared to the control treatment (DMSO). GAPDH was used as a loading control, with respective molecular weights in KDa shown on the right side of each panel. (**E**) Bar graph demonstrating RT-qPCR quantification of *SNAI2*, *SNAI1*, and *TGFβ2* following Dasatinib treatment in three claudin-low cell lines (MDA-MB-231, Hs578T, MDA-MB-436), for 48 h at the respective IC50 concentrations. Expression is presented as a percentage relative to the control (DMSO) and normalized relative to mean of HPRT and SDHA housekeeping genes. *SNAI2* gene encodes Slug, *SNAI1* encodes Snail. Graph represents mean + SD of three independent experiments. *p < 0.05, **p < 0.005, ***p < 0.001. (**F**) Table showing the % relative cell cycle phases (as assayed by flow cytometry) of MDA-MB-436, MDA-MB-231 and Hs578T claudin-low cell lines following treatments with the indicated Dasatinib concentrations for 72 h. (**G**) Western blot analysis of several potential phenotypic markers following treatments with the indicated Dasatinib concentrations for 72 h. GAPDH immunoblotting was used as a loading control

Connectivity Mapping Analysis had highlighted the SRC inhibitor Dasatinib as a potential inhibitor of chemoresistant TNBC, therefore, we explored its utility as an antitumor agent in vitro. Claudin-low TNBC cell lines were more sensitive to Dasatinib than other TNBC cell lines, with colony forming assay experiments showing a reduction in colony number in Dasatinib-treated cells compared with the control, including in the claudin-low cell lines MDA-MB-231, Hs578T and MDA-MB-436 (Fig. 2A). Dose-response experiments also demonstrated that claudin-low cell lines had lower IC50s, of approximately 100nM, compared with non-claudin-low cell lines T47D, MCF7, HCC-1806, MDA-MB-468 and HCC-3153 (Fig. 2B), with claudin-low MDA-MB-436 cells showing dramatically reduced clonogenicity compared to luminal MCF7 cells (Fig. 2C). These findings are in line with a previous report [18] and indicate that Dasatinib is a potent inhibitor of claudin-low TNBC. 

We next explored whether Dasatinib could impact the expression of key mesenchymal and epithelial markers, since claudin-low TNBC is characterized by mesenchymal features and high rates of metastasis. Dasatinib upregulated protein levels of the epithelial marker E-cadherin, and downregulated the mesenchymal markers N-cadherin, Slug and Snail (Fig. 2D). We confirmed that this regulation occurred at the protein stability level, as an RT-qPCR analysis of Dasatinib-treated cells indicated no change in the mRNA levels of *SNAI1* nor *SNAI2*, the genes encoding Snail and Slug. However, *TGFβ2* mRNA was significantly reduced, suggesting that SRC is upstream of *TGFβ2* expression (Fig. 2E). Dasatinib also reduced cell migration as assessed by wound scratch assays (Supplementary Figure S1B) and noticeably changed the morphology of claudin-low cells, giving them an elongated and potentially more epithelial appearance (Supplementary Figure S1C). An xCELLigence experiment confirmed that Dasatinib significantly (*p* < 0.0001) inhibited chemotactic migration and invasion through matrigel (Supplementary Figures S1D-I). Interestingly, investigation by flow cytometry did not indicate an obvious induction of apoptosis in any of the claudin-low cell lines, as evidenced by lack of accumulation of a subG1 population, or of PARP cleavage by western blotting (Fig. 2F and G, respectively). Closer inspection of the redistribution of cell cycle phases following Dasatinib treatment showed a consistent accumulation of cells in the G1 phase in all three claudin-low cell lines, with consistent increases in expression of the quiescence marker p27kip1 across all Dasatinib treatments (Fig. 2G). It therefore appears that inhibition of SRC activity in claudin-low cells has greatest impact on cell migration, invasion, colony formation, and reversal of EMT, rather than induction of apoptosis. In addition, the inhibition of SRC activity induces a quiescence phenotype, which results in marked reduction of cell proliferation as assessed by clonogenic assays.

### Slug is a key driver of poor-outcome TNBC phenotypes and regulates TGFβ2 *via* TGFβ2-AntiSense 1

The downregulation of Slug and Snail protein levels following Dasatinib treatment was particularly intriguing as these transcription factors are well-characterized regulators of the mesenchymal phenotype, and could explain how SRC drives claudin-low TNBC cell survival. Using cBioPortal we explored the relationship between Snail (*SNAI1*) and Slug (*SNAI2*) expression and patient outcomes in the Metabric dataset [7]. In the invasive breast cancer subset, tumors with the highest expression of *SNAI2* had a significantly poorer likelihood of progression-free survival (Supplementary Figure S2A), and high *SNAI2*-expressing tumors were enriched significantly in the claudin-low subtype (Supplementary Figure S2B). Overall, this suggested that Slug signaling may regulate the pathogenesis of poor-outcome breast cancers.

**Fig. 3:**
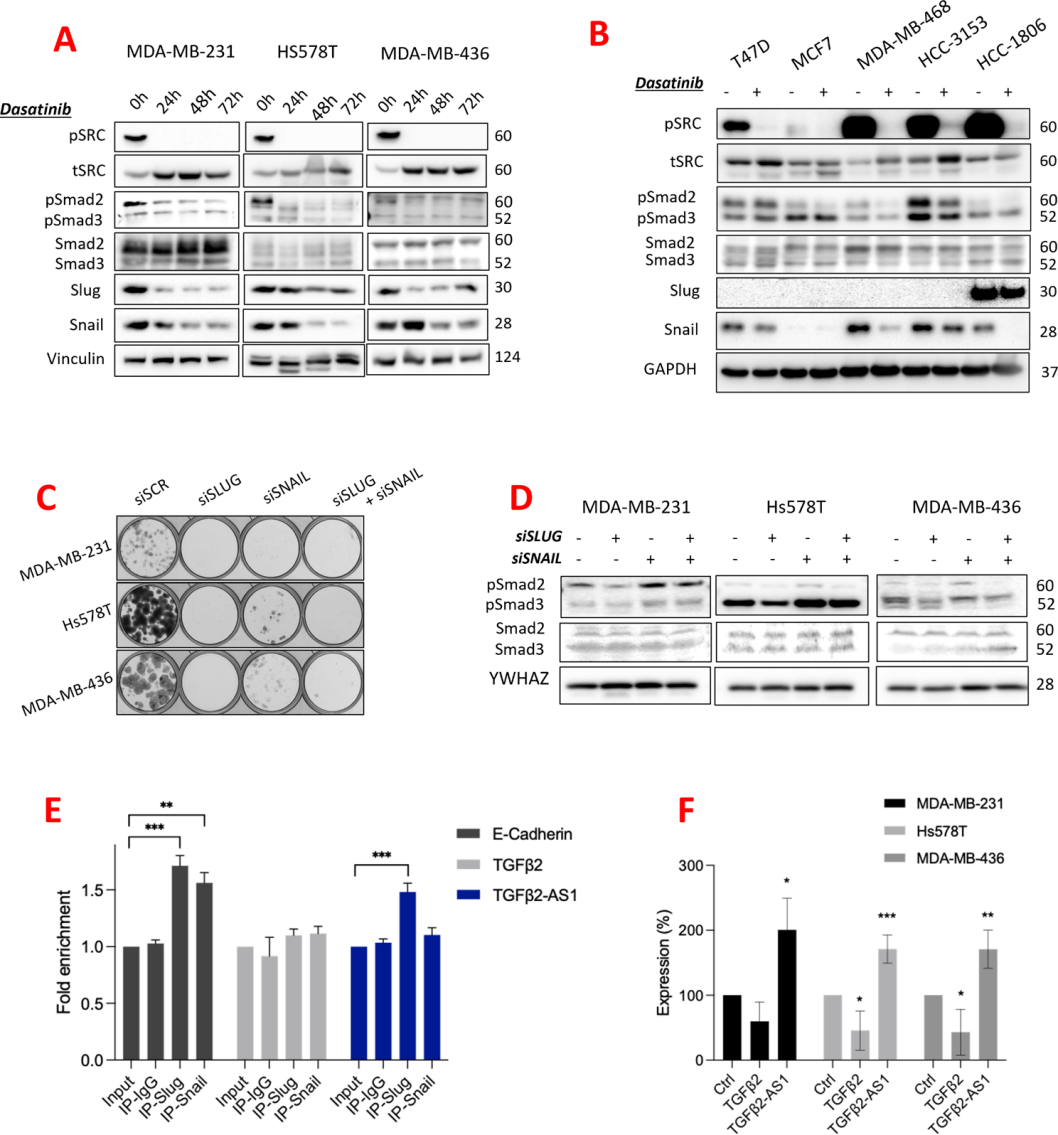
Slug is essential in TNBC and regulates TGFb2 via TGFb2-AS1 expression. (**A**) Western blot analysis of claudin-low cell lines (MDA-MB-231, Hs578T, MDA-MB-436) treated with Dasatinib, at IC50 for up to 72 h. Membranes were immunoblotted for Slug and Snail transcription factors, phosphorylated SRC(Tyr416), total SRC, phosphorylated Smad2(Ser465/467) (pSMAD2) and phosphorylated Smad3(Ser423/425) (pSMAD3), total Smad2/3, with levels measured relative to the control treatment (DMSO). Vinculin was used as a loading control with respective molecular weights in KDa shown on the right side of each panel. (**B**) Western blot analysis of hormone receptor positive cell lines (MCF7 and T47D) and basal-like TNBC cell lines (MDA-MB-468, HCC-3153 and HCC-1806) treated with Dasatinib at the IC50 for 72 h, relative to the control treatment (DMSO). Membranes were immunoblotted for pSMAD2/3, total SMAD2/3, Slug and Snail. GAPDH was used as a loading control with respective molecular weights in KDa shown on the right side of each panel. (**C**) Images of crystal violet stained clonogenic assays 9 days after knockdown of Slug and Snail by siRNA (siSLUG and siSNAIL) in claudin-low cells (MDA-MB-231, Hs578T, MDA-MB-436), compared to the scrambled control siRNA (siSCR). (**D**) Western blot analysis of claudin-low cell lines (MDA-MB-231, Hs578T, MDA-MB-436) following siRNA knockdown of SLUG and SNAIL (siSLUG and siSNAIL). Membranes were immunoblotted for Slug, Snail, phosphorylated SMAD2/3 and total SMAD2/3. YWHAZ is included as a loading control, with respective molecular weights in KDa shown on the right side of each panel. (**E**) Bar graph demonstrating ChIP-PCR data using primers specific to TGFβ2-AS1 and TGFβ2, following pulldown of Slug, Snail, or control antibody (IgG). An E-Cadherin promoter region was included as positive control for Slug and Snail ChIPs. The graph depicts fold enrichment, normalized to expression in the Input sample, and represents the mean + SD of three independent experiments. *p < 0.05, **p < 0.005, ***p < 0.001. (**F**) Bar graph demonstrating RT-qPCR quantification of TGFβ2 and TGFβ2-AS1 in claudin-low cell lines (MDA-MB-231, Hs578T, MDA-MB-436) following knockdown of SNAI2 (siSLUG) relative to the control (siSCR). Expression was normalized relative to mean of HPRT and SDHA housekeeping genes. The graph represents mean + SD of three independent experiments. *p < 0.05, **p < 0.005, ***p < 0.001

Next, we explored further the ability of Dasatinib to downregulate TGFβ signaling. Upon TGFβ receptor stimulation, receptor-regulated SMADs (R-SMADs) are phosphorylated, with phosphorylation levels of SMAD mediators indicative of increased TGFβ signaling. We observed that Dasatinib treatment reduced protein levels of phosphorylated SMAD2 and phosphorylated SMAD3 (pSMAD2/3), without affecting the levels of total SMAD2/3, consistent with a reduction in TGFβ signaling. This correlated with a reduction in Slug and Snail levels also (Fig. 3A). The correlation in the levels of Slug, Snail and pSMAD2/3 was consistent across other breast cancer cell lines, including luminal cell lines (Fig. 3B). Additionally, a colony forming assay experiment revealed that Snail and Slug were essential for the survival of claudin-low cell lines (Fig. 3C). Indeed, other breast cancer cell lines, including luminal cell lines, also displayed a marked survival dependence on Slug (Supplementary Figure S2C), suggesting that this phenotype is not restricted to TNBCs. Interestingly, knockdown of Slug, but not Snail, reduced pSMAD2/3 (Fig. 3D), indicating a Slug-specific TGFβ signaling axis.

We were also interested in defining the mechanism through which Slug regulates *TGFβ2*. ChIP pulldown of Slug revealed that it bound the *TGFβ2* antisense 1 (*TGFβ2*-*AS1*) promoter region (Fig. 3E), in keeping with its well characterized role as a transcriptional repressor [15]. Correspondingly, knockdown of Slug by siRNA increased expression of *TGFβ2*-*AS1* mRNA (Fig. 3F). These observations suggested that Slug represses expression of *TGFβ2*-*AS1*, thereby stabilizing *TGFβ2* mRNA levels and increasing TGFβ2 signaling.

### Regulation of Slug and Snail in claudin-low TNBC cells

**Fig. 4:**
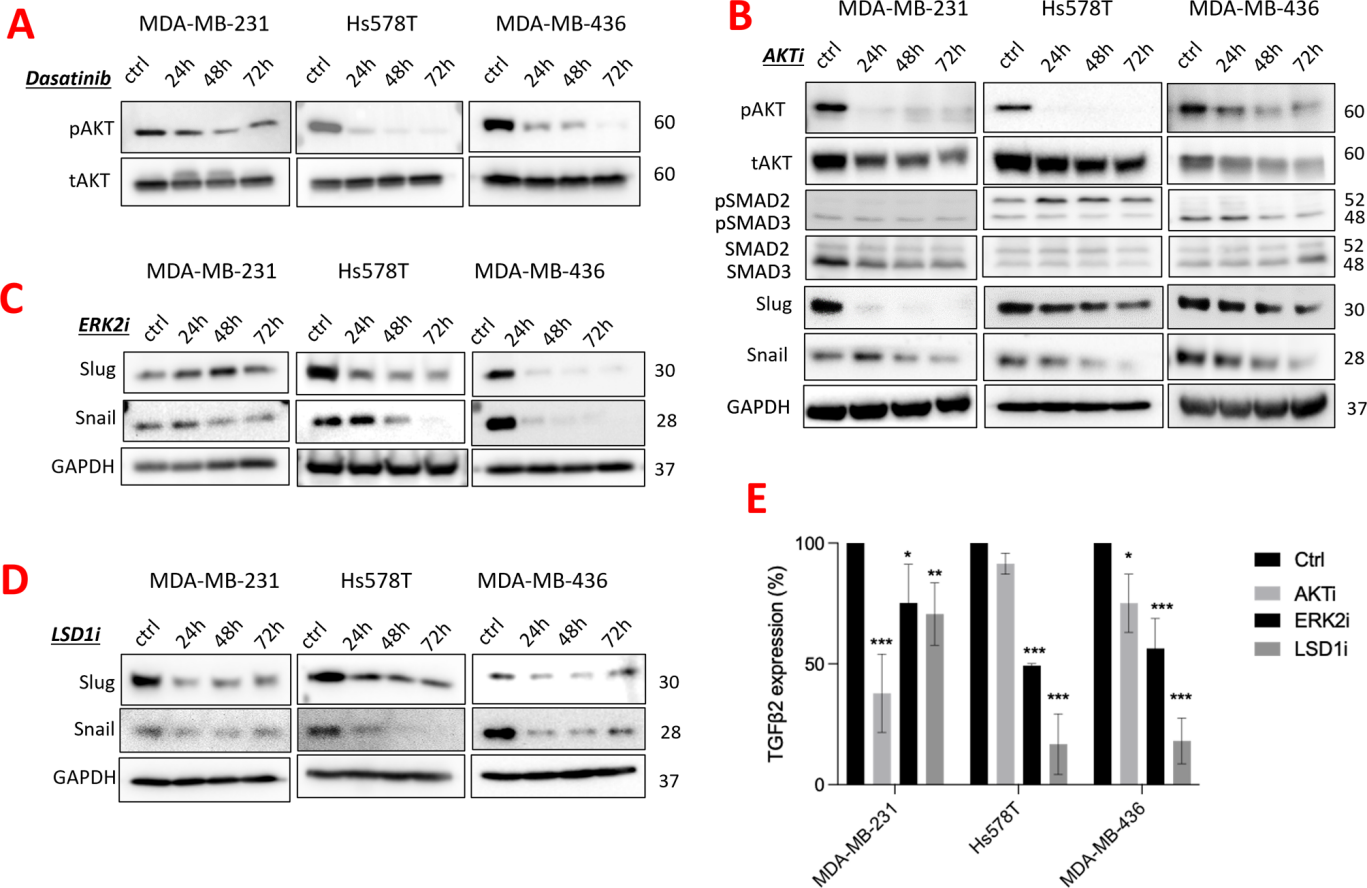
Regulation of Slug and Snail in claudin-low TNBC. (**A**) Western blot analysis of claudin-low cell lines (MDA-MB-231, Hs578T, MDA-MB-436) following Dasatinib treatment (at IC50), with membranes immunoblotted for phosphorylated AKT (Ser473) and total AKT. Respective molecular weights in KDa are shown on the right side of each panel. (**B**) Western blot analysis of claudin-low cell lines (MDA-MB-231, Hs578T, MDA-MB-436) treated with a pan-AKT inhibitor, S7776 (at IC50 for up to 72 h) compared to the control (DMSO). Membranes were immunoblotted for pAKT(Ser473), AKT, phosphorylated SMAD2/3, total SMAD2/3, Slug, and Snail. GAPDH was used as a loading control with respective molecular weights in KDa shown on right side of each panel. (**C**) Western blot analysis showing reduced protein levels of Slug and Snail in claudin-low cell lines (MDA-MB-231, Hs578T, MDA-MB-436) treated with ERK2 inhibitor (VX-11), relative to the control (DMSO). Membranes were immunoblotted for Slug and Snail. GAPDH was used as a loading control with respective molecular weights in KDa shown on the right side of each panel. (**D**) Western blot analysis of claudin-low cell lines (MDA-MB-231, Hs578T, MDA-MB-436) treated with an LSD1 inhibitor (SP-2509), relative to the control (DMSO). Membranes were immunoblotted for Slug and Snail. GAPDH was used as a loading control with respective molecular weights in KDa shown on the right side of each panel. (**E**) Bar graph demonstrating RT-qPCR quantification of claudin-low cell lines (MDA-MB-231, Hs578T, MDA-MB-436) treated with inhibitors of AKT, ERK2 and LSD1 at the IC50, quantifying expression of *TGFβ2*, *SNAI2* and *SNAI1*, and presented as a percentage relative to the control (DMSO). Expression is normalized to the mean of HPRT and SDHA housekeeping genes. Graph represents mean + SD of three independent experiments. *p < 0.05, **p < 0.005, ***p < 0.001

We wanted therefore to investigate the mechanistic basis of regulation of Slug and Snail by SRC. Neither protein possessed a consensus SRC phosphorylation recognition motif, however, SRC was a known upstream regulator of AKT [20], so we hypothesized that AKT could be an intermediary kinase. Accordingly, Dasatinib treatment reduced phosphorylated AKT relative to total AKT (Fig. 4A), and a pan-AKT inhibitor (S7776) downregulated pSMAD2/3, Slug and Snail (Fig. 4B), indicating that SRC regulates Slug and Snail *via* AKT in TNBC. With no obvious or previously reported AKT phosphorylation sites on either Slug or Snail, we subsequently assessed if AKT could indirectly regulate Slug or Snail through its known downstream substrates GSK3β [32], or ERK2 [3], both of which have consensus kinase sites on Slug and Snail. There was no consistent association between Slug or Snail expression with GSK3b (Supplementary Fig. 3A-B) expression following SRC or AKT inhibition, however, ERK2 antagonism with the inhibitor VX-11 reduced Slug and Snail levels (Fig. 4C), thereby implicating ERK2 as another intermediary kinase in SRC stabilization of these EMT transcription factors.

Slug and Snail are known to be comparatively weak DNA-binders and rely for stabilization on target promoters by chromatin modifying enzymes, such as Lysine Demethylase-1 (LSD1) [19]. We were interested to investigate this in the context of TNBC, with the view to characterizing the pathway and identifying additional therapeutic strategies. Both Slug and Snail have been reported to recruit LSD1 through interactions with their amino-terminal SNAG domains, resulting in the establishment of repressive chromatin marks around target promoters, such as E-Cadherin [19]. Accordingly, we observed that treatment of claudin-low cells lines with an LSD1 inhibitor (SP-2509) destabilized both Slug and Snail, indicating that LSD1 may also be required for the stability of these EMT transcription factors in TNBC (Fig. 4D). Indeed inhibitors of enzymes involved in each step of this pathway (AKTi, ERK2i and LSD1i) all reduced *TGFβ2* mRNA (Fig. 4E), further suggesting the presence of a functional SRC-AKT-ERK2-LSD1-Slug-*TGFβ2* signaling axis in TNBC. While Slug was capable of regulating *TGFβ2* expression, it was unidirectional, as knockdown of *TGFβ2* by siRNA did not reduce Slug levels (Supplementary Figure S3C). Inhibitors of AKT, ERK2 and LSD1 all reduced colony forming ability at 1µM or lower, further supporting their roles in the maintenance of TNBC survival through this signalling axis (Supplementary Figure S3D).

### The role of Slug in claudin-low TNBC biology

**Fig. 5:**
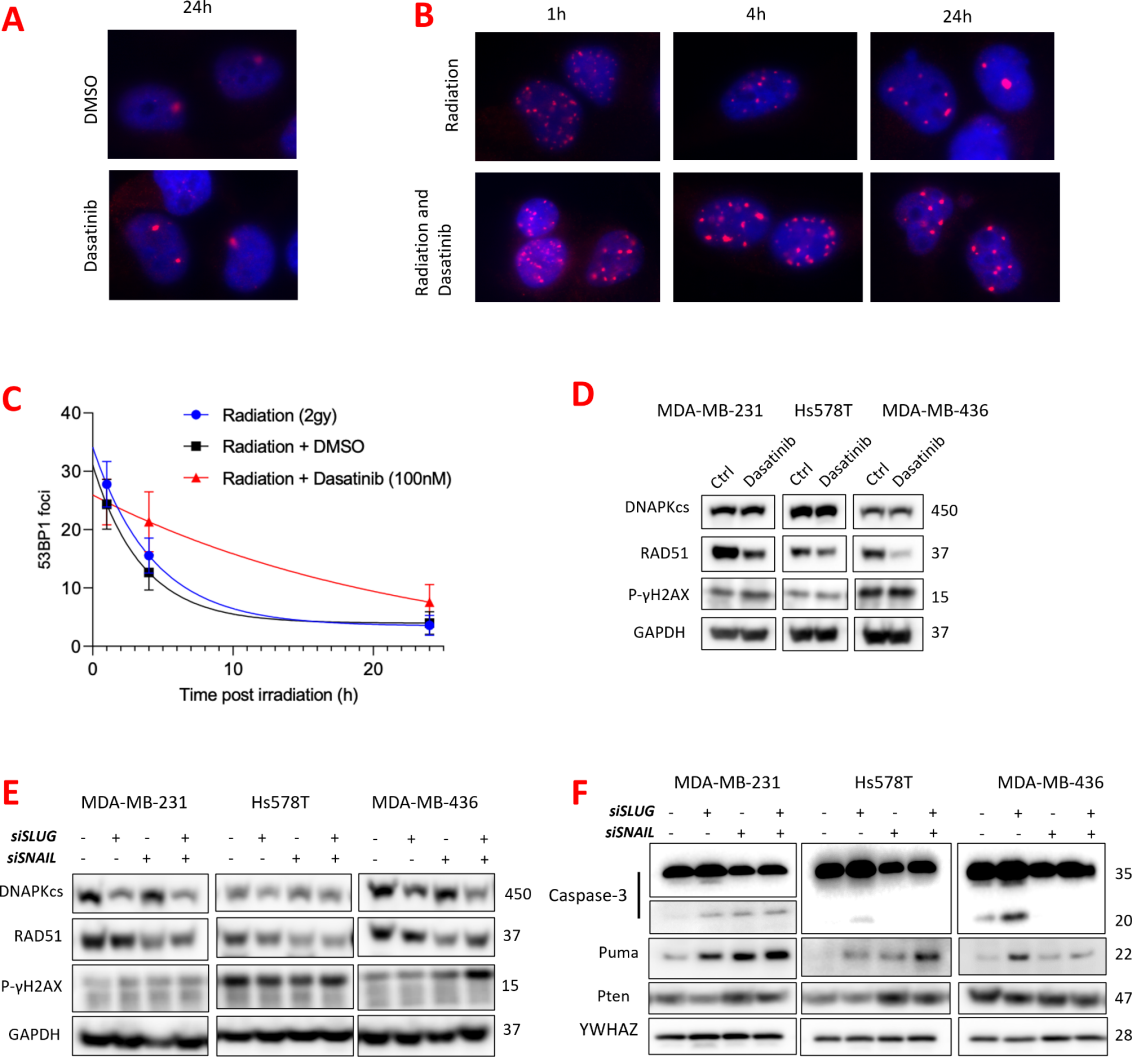
Characterizing the role of Slug in claudin-low TNBC biology. (**A**) Immunofluorescence-based detection of DNA double strand break repair foci in MDA-MB-436 cells after 24 h of Dasatinib treatment, compared with the control condition (DMSO). Blue stain represents DAPI-stained nuclei, red stain depicts 53BP1 foci. (**B**) Immunofluorescence-based detection of DNA double strand break repair foci in MDA-MB-436 cells treated for 24 h with Dasatinib at the IC50 and then subjected to ionising radiation (2gy). Left-hand panels demonstrate double-strand break foci 1 h following radiation, and then 4 h and 24 h post irradiation. The combination of Dasatinib and radiation is compared with the control condition (radiation and DMSO). Blue stain represents DAPI-stained nuclei, red stain depicts 53BP1 foci. (**C**) Line graph demonstrating the repair kinetics of the radiation-induced 53BP1 foci following ionising radiation (2gy), with cells pre-treated with Dasatinib (at IC50 for 48 h), relative to control (DMSO). Points represent the mean number of foci per cell of three independent experiments and the respective standard error. Data were corrected for the baseline mean foci value and fitted to an exponential decay equation. *p < 0.05, **p < 0.005, ***p < 0.001. (**D**) Western blot analysis of claudin-low cells (MDA-MB-231, Hs578T, MDA-MB-436) treated with Dasatinib at the IC50 compared to the control (DMSO). Membranes were immunoblotted for DNAPKcs, RAD51 and phosphorylated γH2AX (Ser139). GAPDH was used as a loading control with respective molecular weights in KDa shown on the right side of each panel. (**E**) Western blot of claudin-low cells with knockdown of Slug (siSLUG) and Snail (siSNAIL) compared to the control (siSCR). Membranes were immunoblotted for phosphorylated γH2AX (ser139), RAD51, and DNAPKcs. GAPDH was used as a loading control with respective molecular weights in KDa shown on the right side of each panel. (**F**) Western blot analysis of claudin-low cells (MDA-MB-231, Hs578T, MDA-MB-436) with siRNA knockdown of Slug (siSLUG) and Snail (siSNAIL) compared to the control (siSCR). Membranes were immunoblotted for Caspase-3, phosphorylated SMAD2/3, total SMAD2/3, Puma and Pten. YWHAZ was used as a loading control with respective molecular weights in KDa shown on the right side of each panel

We next explored the function of Slug and Snail in driving poor outcomes in claudin-low TNBC. We focused on Slug as it was previously reported to mediate DNA double strand break (DSB) repair in non-transformed MCF10A breast cells [12]. Similarly, we observed in the TNBC setting, a significantly impaired rate of DSB repair in Dasatinib-treated claudin-low cells following ionizing radiation. Dasatinib treatment alone did not induce DNA-damage (Fig. 5A) but it reduced the rate of repair of DSBs induced by ionizing radiation (Fig. 5B-C). However, unlike in the MCF10A cell study [12], Dasatinib treatment also reduced the protein levels of the homologous recombination (HR) mediator RAD51, but not the non-homologous end joining (NHEJ) mediator DNAPKcs, specifically highlighting a role in HR (Fig. 5D). Knockdown of Slug and Snail by siRNA suggested that Snail may regulate RAD51 and Slug may regulate DNAPKcs (Fig. 5E). *RAD51* mRNA expression levels were unaffected by Dasatinib treatment (Supplementary Figure S4A), suggesting Dasatinib reduces RAD51 protein stability rather than expression.

Since Slug and Snail were essential for TNBC cell survival, we explored whether this was apoptosis-dependent. Knockdown of Slug by siRNA (siSLUG) induced caspase-3 cleavage and expression of known Slug-repressed targets, such as the pro-apoptotic protein PUMA (and to a lesser extent PTEN), indicating a Slug role in anti-apoptosis signaling (Fig. 5F). However, treatment with the caspase-3 inhibitor Z-VAD was not sufficient to rescue the effects of siSLUG, or Dasatinib (Supplementary Fig. 4B-C), suggesting the potential contribution of other survival axes. Considering the potential role of Slug/Snail in DNA repair, we assessed the potential for drug synergy between Dasatinib and the PARP inhibitor, Olaparib. However, and unlike previous reports [4], we did not detect synergy in vitro on cell viability or colony forming assay (data not shown). Finally, a flow cytometry analysis confirmed that siRNA knockdown of Slug reduced the number of cells in G1 phase in the Hs578T and MDA-MB-436 cell lines, but not MDA-MB-231 cells (Supplementary Fig. 4D), suggesting that Slug may also regulate cell proliferation, although additional contributory signaling may be required.

**Fig. 6:**
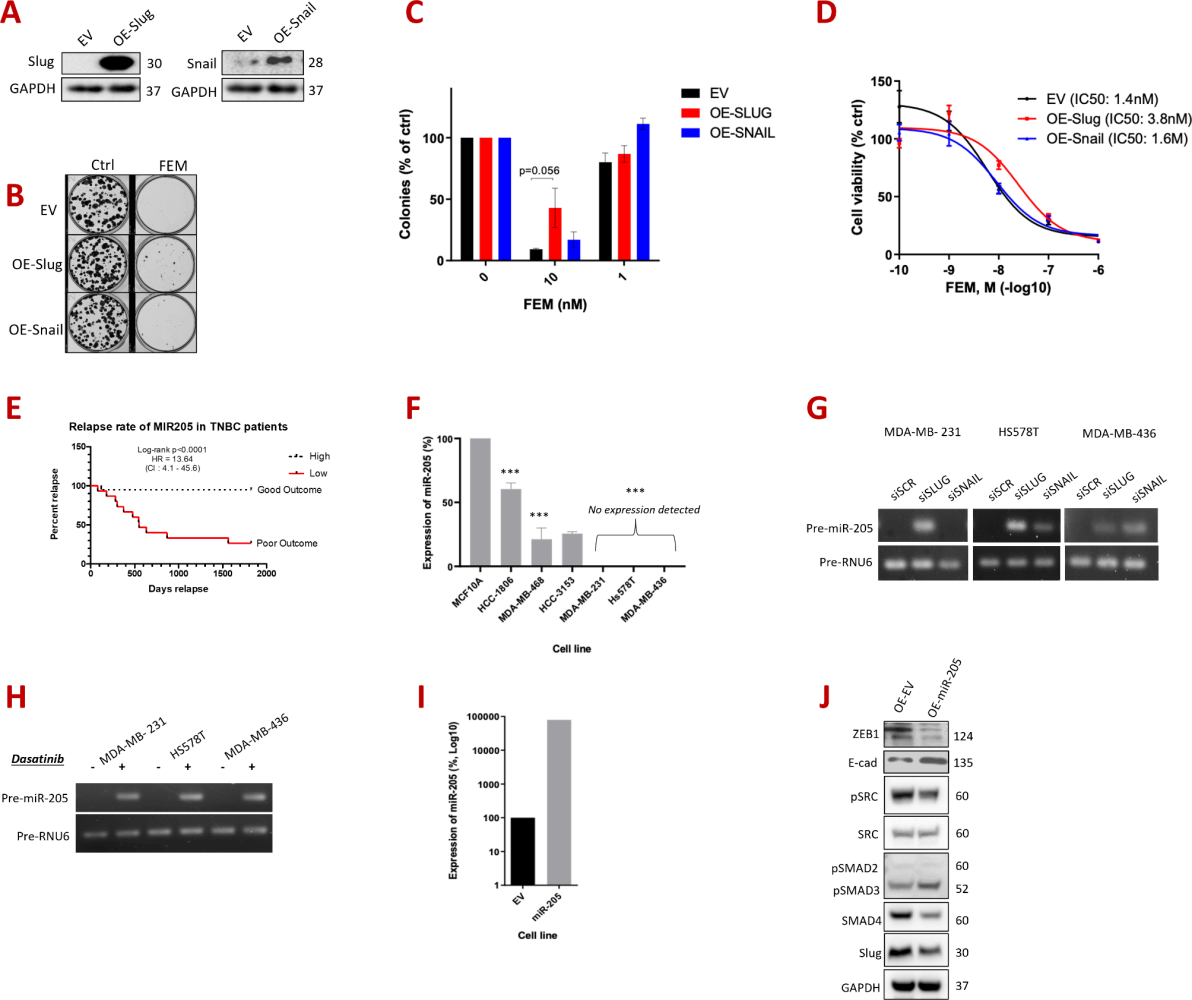
Exploration of the role of Slug and Snail in modulating chemoresistance in claudin-low TNBC. (**A**) Western blot analysis of basal-like TNBC cell line (MDA-MB-468) with ectopic overexpression of Slug and Snail, compared to an empty vector (EV) control overexpression. Membranes were immunoblotted for Slug and Snail, with GAPDH included as a loading control and with respective molecular weights in KDa shown on the right side of each panel. (**B**) Images of clonogenic assays of MDA-MB-468 cells with overexpression of EV, Slug or Snail, 9 days following treatment with either FEM chemotherapy, or control treatment (DMSO). (**C**) Bar graph depicting the clonogenicity values of cells from panel B (namely, MDA-MB-468 with overexpression of EV, Slug or Snail), 9 days following treatment with FEM or control (DMSO). (**D**) Line graph depicting the dose response analysis of FEM (measured by MTT viability assay) in MDA-MB-468 with overexpression of EV, Slug, or Snail. (**E**) Bar graph demonstrating RT-qPCR quantification of pre-miR-205 in basal-like TNBC cell lines (MDA-MB-468, HCC-1806 and HCC-3153) and claudin-low cell lines (MDA-MB-231, Hs578T, MDA-MB-436), relative to non-transformed (MCF10A). Expression normalized to the mean of GAPDH and HPRT housekeeping genes. Graph represents mean + SD of three independent experiments. *p < 0.05, **p < 0.005, ***p < 0.001. (**F**) Kaplan-Meier plot representing survival in TNBC patients stratified by miR-205expression (KM Plotter).(**G**) Semi-quantitative PCR of pre-miR-205 expression in claudin-low cells (MDA-MB-231, Hs578T, MDA-MB-436) with knockdown of Slug (siSLUG) and Snail (siSNAIL). Expression was quantified relative to pre-miR-RNU6. (**H**) Semi-quantitative PCR of pre-miR-205 expression in claudin-low cells (MDA-MB-231, Hs578T, MDA-MB-436) treated with Dasatinib (at the IC50 for 72 h). Expression was quantified relative to pre-miR-RNU6. (**I**) Bar graph illustrating RT-qPCR quantification of pre-miR-205 (miR-205) in Hs578T cells with exogenous overexpression of pre-miR-205, relative to the control Hs578T cells overexpressing the empty vector (EV). Expression is normalized to mean of SDHA and HPRT housekeeping genes. Both cell lines were generated as mixed populations. (**J**) Western blot analysis of Hs578T cells with exogenous overexpression of pre-miR-205 compared with EV-overexpressing Hs578T. Membranes were immunoblotted for ZEB1, E-cadherin (E-cad), pSRC, SRC, pSMAD2/3, SMAD4, and Slug. GAPDH was included as a loading control, with respective molecular weights in KDa shown on the right side of the panel

We next examined if Slug or Snail mediated the chemoresistance of claudin-low cells. No synergy was identified between Dasatinib and FEM chemotherapy in vitro. However, ectopic overexpression of Slug in the chemosensitive, basal-like TNBC cell line, MDA-MB-468, increased its chemoresistance, with clonogenic assays showing that IC50 values for FEM were doubled, relative to empty vector control transfected MDA-MB-468 cells (Fig. 6A-D).

We also identified the epithelial miRNA, miR-205, as a Slug target. MiR-205 is well-established as a tumor suppressor in multiple cancer types including in breast cancer, regulating key EMT factors including ZEB1/2 [11]. MiR-205 was shown to be reduced in breast cancer relative to normal breast tissue and is further reduced in metastatic TNBC [43], and a KM Plotter analysis indicated that higher expression of miR-205 in tumors positively correlated with survival (Fig. 6E). Similarly, the precursor molecule *pre-miR-205* is expressed at lower levels in basal-like TNBC cell lines relative to non-transformed HME1 cells, with expression virtually undetectable in claudin-low cells (Fig. 6F). In line with these indications that miR-205 is a tumor suppressor in TNBC, we observed that siRNA knockdown of Slug, or treating claudin-low cells with Dasatinib, both upregulated the expression of *pre-miR-205* (Fig. 6G-H). To study the functional impact of miR-205 in counteracting this TGFβ2-SRC-Slug signaling axis, we generated a cell line derived from claudin-low Hs578T cells, with constitutive expression of *pre-miR-205* (Fig. 6I). We observed that the TGFβ signaling proteins SMAD4 and ZEB1 (both known miR-205 targets) were reduced, whilst *E-Cadherin* expression was increased (Fig. 6J). Using online prediction tools (such as the TransmiR v2.0 database), Slug and SRC were also predicted to be potential miR-205 targets [42]. Accordingly, protein levels of Slug and SRC were reduced in the miR-205 overexpressing Hs578T cells (Fig. 6I). Therefore, miR-205 loss may be a key event in poor-outcome TNBC biology, where it acts as a negative regulator of TGFβ2-SRC-Slug signaling, with reciprocal Slug repression of miR-205 enforcing an EMT-like, chemoresistant, pro-metastatic loop.

**Fig. 7:**
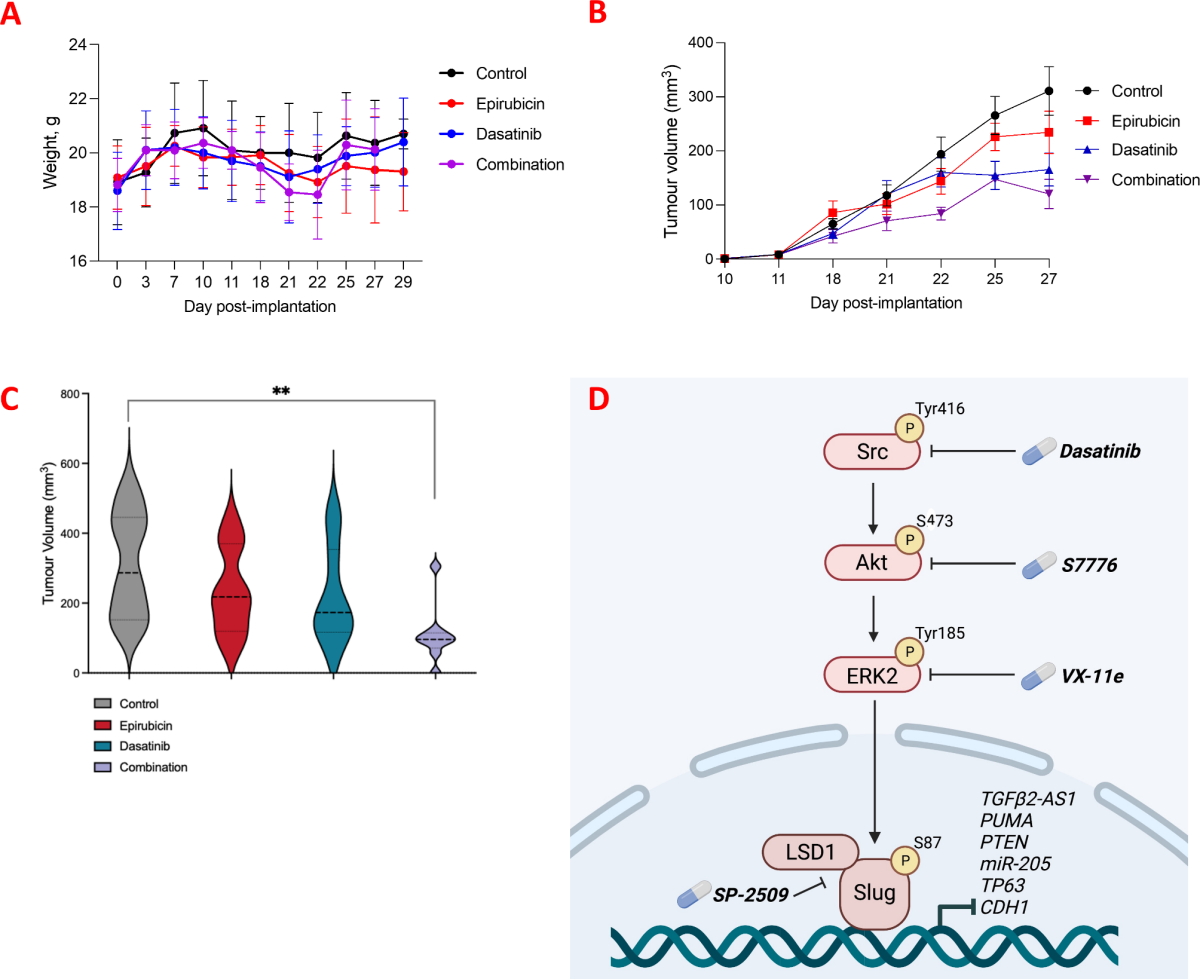
In vivo exploration of Dasatinib in allograft orthotopic model of claudin-low TNBC. (**A**) Line graph depicting mouse weights in grams throughout the duration of the study by treatment group (Epirubicin, Dasatinib, and a combination of Epirubicin and Dasatinib, all expressed relative to the Control, DMSO), and shown as mean + SD. *N* = 6 per group. (**B**) Line graph depiction of tumor volumes (mm^3^) throughout the duration of the study by treatment group (Epirubicin, Dasatinib, and a combination of Epirubicin and Dasatinib, relative to the Control, DMSO), and shown as mean + SD. *N* = 6 per group. (**C**) Box and violin plot of tumor volumes (mm^3^) by treatment group (DMSO control, Epirubicin, Dasatinib, and a combination of Epirubicin and Dasatinib) at the end of the study (day 29 post-implantation). *p < 0.05, **p < 0.005, ***p < 0.001. (**D**) Diagrammatic depiction of the hypothesized pathway driving poor-outcome TNBC, where SRC signals *via* AKT and ERK2 to stabilise the Slug/Snail transcription factor complex (including LSD1), which in turn represses expression of *TGFβ2-AS1* and other tumor suppressors (such as PTEN, PUMA, etc.). Some suggested points of intervention with inhibitory drugs are shown

Finally, the potential use of Dasatinib to modulate the pathway and tumor development in vivo was explored in an allograft orthotopic, immunocompetent model. We used the murine line 4T1, after confirming that it displayed the mesenchymal hallmarks observed in human claudin-low TNBC cells, including a reliance on SRC signaling for regulation of migration, pAKT, and levels of Slug and Snail (Supplementary Figure S5A-B). We used young female balb/c mice to represent the propensity of TNBC to affect younger women. The objective was to determine the ability of Dasatinib alone, or in combination with the anthracycline, Epirubicin (which is almost invariably included in the TNBC treatment regimen), to impair tumor development. We established orthotopic allografts, and treated mice with Dasatinib, Epirubicin, the combination of both, or the control (vehicle). Dasatinib and the combination was very well tolerated, with no adverse effects or weight loss (Fig. 7A). Dasatinib reduced tumor volume better than Epirubicin, and the combination of both was more effective than either drug individually (Fig. 7B-C). Figure 7D illustrates the hypothesized pathway driving pathogenesis in claudin-low TNBC. We propose that SRC and Slug act as effectors enforcing the repression of key anti-EMT regulators (such as miR-205 and Puma), thereby establishing an aggressive signaling axis which drives chemoresistance and metastases. We also point out the novel therapeutic opportunities which can now be utilised by targeting the members of this pathway.

## Discussion

It is well documented that most TNBCs are intrinsically resistant, or acquire resistance to chemotherapy regimens like FEC, and the lack of confirmed driver oncogenic pathways has made this subtype a key area of unmet clinical need in breast cancer. We generated a differential signature of poor-outcome TNBC, through stratifying gene expression profiles based on clinical outcome, and identified TGFβ2 and SRC signaling as two highly upregulated signaling nodes. Our in vitro experiments then demonstrated that SRC is an upstream regulator of *TGFβ2* in TNBC, *via* AKT, ERK2, LSD1 and the Slug and Snail transcription factors. We also showed for the first time that Slug regulates TGFβ2 *via* transcriptional repression of its antisense RNA, *TGFβ2-AS1*. We conclude that this signaling axis could be activated in poor-outcome TNBC, and may contribute to survival and chemoresistance.

In line with the known role of TGFβ2, our in vitro knockdown of *TGFβ2* impacted on migration and invasion rather than proliferation, and it was particularly important in the in vivo tumor setting. In keeping with a role in driving poor outcome TNBC biology, TGFβ2 has been previously shown to dictate disseminated tumor cell (DTC) fate in target organs [1]. TGF-β2 specifically signals through TGF-β-RIII - p38α/β, to induce DTC dormancy and potentially render DTCs unresponsive to systemic chemotherapies (such as those currently used in TNBC treatments). Our in vitro experiments also implicated Slug as a key driver of claudin-low TNBC. This is in keeping with Slug’s known roles in regulating stemness and ‘plasticity’. For example, ChIP analyses in the luminal breast cancer line MCF7 following ectopic expression of Slug produced distinct lineage reprogramming, with transcriptional upregulation of genes associated with the claudin-low phenotype, and equally transcriptional repression of luminal genes (29). Increased cell migration conferred by Slug overexpression was also abolished by a TGFβ inhibitor [5]. Slug, along with Sox9, was reported to be the predominant EMT transcription factor expressed in mammary stem cells, and ectopic expression also conferred stem cell attributes to differentiated mammary epithelial cells [14]. *SNAI2* mutant mice exhibited a decline in mammary stem cell activity and a reduced capacity to develop breast cancers [29]. Slug was also reported a master regulator of the differentiation status of epidermal progenitor cells [24]. Poor-outcome TNBC tumors also displayed lower levels of *TGFβ2*-*AS1* [48], in line with our ChIP results. A systematic review and meta-analysis concluded that elevated Slug protein expression may be related to poor outcomes in patients with breast cancer. Increased Slug expression was associated with a higher TNM stage and a higher likelihood of axillary lymph node metastasis, indicating its potential as an indicator of patient survival and a new target for breast cancer therapy [46]. Our data adds to the evidence for Slug as a key driver of poor outcome breast cancers, and specifically implicates it in the TNBC subtype.

We explored the role of Slug in several pathways, and conclude that it could be involved in several survival axes: driving anti-apoptosis signaling, and facilitating chemoresistance. Slug was previously shown to facilitate DNA damage repair in MCF10A cells following ionising radiation or replicative stress [12]. Accordingly, we observed that treating claudin-low cells lines with Dasatinib reduced the rate of DNA repair following ionising radiation, accompanied by reduced RAD51 expression. We hypothesise that SRC hyperactivity (and consequently increased Slug and Snail stability) could improve TNBC cells’ capacities to survive during the high rates of DNA damage that occur during tumor development, as well as following chemotherapy treatments. However, knockdown of Slug or Snail individually had differing effects on the expression levels of key DNA repair genes, such as DNA-PKcs and RAD51. The roles of these TFs in controlling the expression of DNA repair genes are likely to be gene-specific and context-dependent. Inhibition of SRC through Dasatinib treatment would likely induce more profound effects on DNA repair, through the destabilisation of both TFs simultaneously. Together these findings highlight the diverse range of essential processes that Slug and Snail may regulate in the context of claudin-low TNBC. Further exploration of their transcriptional targets and signaling networks (for instance by ChIP-seq), would be useful to elucidate further their individual and combined functions.

While transcription factors are notoriously difficult to target therapeutically, we demonstrated that Slug could be destabilized in TNBC using low doses of inhibitors of SRC, AKT, ERK2, and LSD1. AKT inhibitors are currently in Phase III clinical trials for TNBC, since the tumors frequently display PI3K/AKT signaling hyperactivation due to *PIK3CA* or *AKT1* mutations and/or *PTEN* inactivation [21]. Our data could suggest an additional mechanism for the efficacy of AKT inhibition in the context of claudin-low TNBC. These results may also have clinical value for other cancer types, such as colorectal cancer which often display overexpression of Slug [35], or clear cell renal carcinoma which was recently shown to contain a claudin-low subtype [47].

To target Slug in vivo we focused on Dasatinib since it has been well tolerated by cancer patients alone or in conjunction with chemotherapy [6]. Dasatinib (Sprycel) is an orally available, clinically approved drug used to treat chronic myeloid leukaemia or Philadelphia chromosome–positive acute lymphoblastic leukaemia [16]. A generic version (Dasatinib Accord) was approved in the EU in March 2022, so it is therefore cost-effective, and would be more accessible worldwide than other new treatments for TNBC such as immunotherapy [45]. As a single agent, Dasatinib has had limited efficacy in Phase I clinical trials. In one study, advanced breast cancer patients with bone metastases were recruited, with ‘SRC responsiveness’ gene signatures (derived from cell line studies) and were treated with Dasatinib, but only one experienced a clinical benefit [31]. However, these tumors could have been enriched in basal-like tumors where SRC signaling is also highly active. Dasatinib treatment in patients with bone metastasis (where only a small fraction were TNBC patients) revealed no significant overall effect on PFS [33]. Another clinical trial of metastatic TNBC was halted due to disease progression in 26 out of 44 patients (ClinicalTrials.gov identifier: NCT00371254). An efficacy study in 2009 in 22 neoadjuvant locally advanced TNBC patients was terminated due to futility after an interim analysis (2 patients had a partial response, 15 had stable disease and 5 had disease progression after 3–4 weeks of 100 mg Dasatinib once daily)(ClinicalTrials.gov identifier: NCT00817531). Overall, this suggests that Dasatinib is not effective as a single agent in breast cancer. However, it is important to consider that the perpetual caveat of clinical trials is the use of metastatic and generally heavily pre-treated cancer patients in typically small cohorts, often with little stratification based on the underlying disease biology (for example, based on elevated TGFβ2 or Slug expression, as alluded to in this study). Moreover, there have been some encouraging results in trials assessing the utility of Dasatinib in combination with other agents. In a Phase I study of Dasatinib and Paclitaxel, 4/14 patients treated displayed a partial response, although it was not stated whether any were TNBC [6]. This justified a Phase II trial including 20% TNBC patients, where one TNBC patient experienced a complete response. The overall response rate was 23%, failing to meet the predefined cutoff of 30% and thus not deemed worthy of future study, although the authors noted that three out of the eight patients with disease response had previously received Paclitaxel, suggesting at least some activity for Dasatinib [25]. A Phase I trial of Dasatinib plus Capecitabine in advanced breast cancer reported a clinical benefit in 56% of response-evaluable patients, although patients with HR positive breast cancers experienced enhanced effects compared with TNBC [36]. There was also a case report of a patient with metastatic TNBC, where the clinicians speculated that the addition of Dasatinib may have contributed to the unexpectedly good response on her breast cancer metastases [34]. With this in mind, we included a combination of Dasatinib and the chemotherapeutic Epirubicin in the in vivo study, and we observed that Dasatinib effectively impeded tumor development when combined with Epirubicin.

In summary, our data suggests that poor-outcome, claudin-low TNBC is critically dependent on SRC and Slug-driven signaling. To our knowledge, no clinical trial targeting Slug in breast cancer has selected patients with mesenchymal or claudin-low molecular markers. This study provides evidence to assist the future stratification of TNBC patients, to ensure that poor-outcome TNBCs can be identified earlier, and suggests that the use of Slug and TGFβ2 modulators could be included in future as part of a treatment regimen for poor-outcome TNBC, to improve the poor survival outcomes associated with this highly aggressive subtype of breast cancer.

## Electronic supplementary material

Below is the link to the electronic supplementary material.

Supplementary Material 1

Supplementary Material 2

Supplementary Material 3

Supplementary Material 4

Supplementary Material 5

Supplementary Material 6

Supplementary Material 7

Supplementary Material 8

Supplementary Material 9

Supplementary Material 10

## Data Availability

No datasets were generated or analysed during the current study.

## Abbreviations

TNBC: Triple-Negative breast cancer
FEC: The chemotherapy cocktail of 5-Fluorouracil, Epirubicin and Cyclophosphamide (FEC) given as standard of care to TNBC patients in the UK
FEM: The chemotherapy cocktail of 5-Fluorouracil, Epirubicin and Mitomycin C (FEM) used in vitro, where mitomycin C is the in vitro substitute for Cyclophosphamide
PCA: Principal Component Analysis
EMT: Epithelial to Mesenchymal Transition
TGFβ2-AS1: Transforming Growth Factor beta-2 (TGFβ2) AntiSense 1
DDR: DNA-damage response
DSB: Double strand break
HR: Homologous recombination
NHEJ: Non-homologous end joining
