# Supplementary material for: A SRC-slug-TGFβ2 signaling axis drives poor outcomes in triple-negative breast cancers
Source: Cell Commun Signal. 2024 Sep 26;22:454. doi: 10.1186/s12964-024-01793-6 (PMC11426005; doi:10.1186/s12964-024-01793-6)
Supplement: Supplementary file 1 — Supplementary Material 1 [file 12964_2024_1793_MOESM1_ESM.docx]

### Appendix 1 -Drugs

| **Name** | **Catalogue number** | **Manufacturer** |
| --- | --- | --- |
| Dasatinib | SML2589 | Sigma (via Merck) |
| Akti-1/2 | S7776 | Selleckchem |
| SP-2509 | n/a | Produced in-house by Rich Williams, QUB |
| Resistomycin | BVT-0036-M001 | Adipogen Life Sciences |
| FEM | n/a | Sourced from Northern Ireland Cancer Centre |
| ERK2I (VX-11) | S7709 | Selleckchem |
