# Supplementary material for: A SRC-slug-TGFβ2 signaling axis drives poor outcomes in triple-negative breast cancers
Source: Cell Commun Signal. 2024 Sep 26;22:454. doi: 10.1186/s12964-024-01793-6 (PMC11426005; doi:10.1186/s12964-024-01793-6)
Supplement: Supplementary file 2 — Supplementary Material 2 [file 12964_2024_1793_MOESM2_ESM.docx]

### Appendix 2 – PCR primers

| **Real-Time PCR** |  |  |
| --- | --- | --- |
| **HUMAN** | **FW (5'3')** | **RV (5'-3')** |
| TGFB2 | ACAACACCCTCTGGCTCAGT | TAGAAAGTGGGCGGGATG |
| SNAI1 | CAGTGCCTCGACCACTATGC | TGCTGGAAGGTAAACTCTGGAT |
| SNAI2 | TCGGACCCACACATTACCTTG | AAAAGGCTTCTCCCCCGTGT |
| B-ACTIN | AGACCTGTACGCCAACACAG | GGAGCAATGATCTTGATCTTCA |
| GAPDH | ACCACAGTCCATGCCATCAC | TCCACCACCCTGTTGCTGTA |
| HPRT | TCACCTTGATTTATTTTGCATACC | CGAGCAAGACGTTCAGTCCT |
| SDHA | CCTGTCCTATGTGGACGTTG | GTTTTGTCGATCACGGGTCT |
| MIR-205 | CCTTCATTCCACCGGAGT | GAACTTCACTCCACTGAAATCTG |
| RNU6 | CTCGCTTCGGCAGCACA | AAC GCT TCA CGA ATT TGC GT |
| RAD51 | ATGGAGTCTTGTGCCAAACC | CTCCCAAAGTGCTGGGAGTA |
| DNAPKcs | GGGGCATTTCCGGGTCC | GACAAGGTCTCCTGCAGCC |
| TGFB2-AS1 | AGGGAGTGTGGAAATGAGG | GGGTTTGGGAGTACATTCAAC |
|  |  |  |
| **MURINE:** | **FW (5'3')** | **RV (5'-3')** |
| SNAI1 | TAGGTCGCTCTGGCCAACAT | CTGGAAGGTGAACTCCACACA |
| SNAI2 | ACTGGACACACACACAGTTAT | TGCCGACGATGTCCATACAG |
| TGFB2 | TCCCCTCCGAAAATGCCATC | ACTCTGCCTTCACCAGATTCG |
| B-ACTIN | CACTGTCGAGTCGCGTCC | TCATCCATGGCGAACTGGTG |
| MIR-205 | ACGCTGCAGGTGCTTTTAGT | CTCCTTCAGGTCACACAGCA |
| RNU6 | CGCTTCGGCAGCACATATAC | AAAATATGGAACGCTTCACGA |
| BCL2 | GAACTGGGGGAGGATTGTGG | GCATGCTGGGGCCATATAGT |
| PTEN | AGCCTCTTGATGTGTGCATT | CCATTGGTAGCCAAACGGAAC |
| PUMA | AGGTGCCTCAATAGCAACCC | CTCCCTGGAGCCCCG |

| **ChIP-PCR** |  |  |
| --- | --- | --- |
|  | **FW (5'3')** | **RV (5'-3')** |
| E-Cadherin | AGCTTGCGGAAGTCAGTTCA | AATGCGTCCCTCGCAAGTC |
| TGFB2 | TCTTGGCCCCATTTCAGAGC | GCACCTGCAGTGCCTGTTTA |
| TGFB2-AS1 | CCTCCTTCCTCCCTTACCCA | TCTCTGAACCACGTGTCTGC |
