# Supplementary material for: A SRC-slug-TGFβ2 signaling axis drives poor outcomes in triple-negative breast cancers
Source: Cell Commun Signal. 2024 Sep 26;22:454. doi: 10.1186/s12964-024-01793-6 (PMC11426005; doi:10.1186/s12964-024-01793-6)
Supplement: Supplementary file 3 — Supplementary Material 3 [file 12964_2024_1793_MOESM3_ESM.docx]

### Appendix 3 – Antibodies

| **Target** | **Species** | **Catalogue Number** | **Manufacturer** | **Dilution** |
| --- | --- | --- | --- | --- |
| Slug (C19G7) | Rabbit mAb | 9585 | Cell Signaling Technology | 1:500 |
| Snail (C15D3) | Rabbit mAb | 3879 | Cell Signaling Technology | 1:500 |
| Phospho-Smad2 (Ser465/467)/Smad3 (Ser423/425) (D27F4) | Rabbit mAb | 8828 | Cell Signaling Technology | 1:500 |
| SMAD2/3 | Goat polyclonal | AF3797 | R&D Systems | 1:500 |
| Phospho-Src Family (Tyr416) | Rabbit mAb | 2101 | Cell Signaling Technology | 1:1000 |
| Src (36D10) | Rabbit mAb | 2109 | Cell Signaling Technology | 1:1000 |
| Phospho-Akt (Ser473) | Rabbit polyclonal | 9271 | Cell Signaling Technology | 1:1000 |
| Akt (pan) (11E7) | Rabbit mAb | 4685 | Cell Signaling Technology | 1:1000 |
| E-Cadherin (24E10) | Rabbit mAb | 3195 | Cell Signaling Technology | 1:1000 |
| N-Cadherin (D4R1H) | Rabbit mAb | 13116 | Cell Signaling Technology | 1:1000 |
| Puma (E2P7G) | Rabbit mAb | 98672 | Cell Signaling Technology | 1:1000 |
| PTEN | Rabbit polyclonal | 9552 | Cell Signaling Technology | 1:1000 |
| EGFR | Mouse mAb | 89414 | BD Transduction | 1:1000 |
| Recombinant Anti-p63 antibody [EPR5701] | Rabbit mAb | ab124762 | Abcam | 1:500 |
| GAPDH (6C5) | Mouse mAb | sc-32233 | Santa Cruz Biotechnology | 1:1000 |
| Vinculin | Rabbit polyclonal | 4650 | Cell Signaling Technology | 1:1000 |
| β-Tubulin | Rabbit polyclonal | 2146 | Cell Signaling Technology | 1:1000 |
| Phospho-GSK3B | Rabbit polyclonal | 9336 | Cell Signaling Technology | 1:1000 |
| VE-Cadherin | Rabbit monoclonal | MA5-29141 | Thermo Fisher Scientific | 1:1000 |
