# Supplementary material for: A SRC-slug-TGFβ2 signaling axis drives poor outcomes in triple-negative breast cancers
Source: Cell Commun Signal. 2024 Sep 26;22:454. doi: 10.1186/s12964-024-01793-6 (PMC11426005; doi:10.1186/s12964-024-01793-6)
Supplement: Supplementary file 4 — Supplementary Material 4 [file 12964_2024_1793_MOESM4_ESM.pptx]

## Slide 1
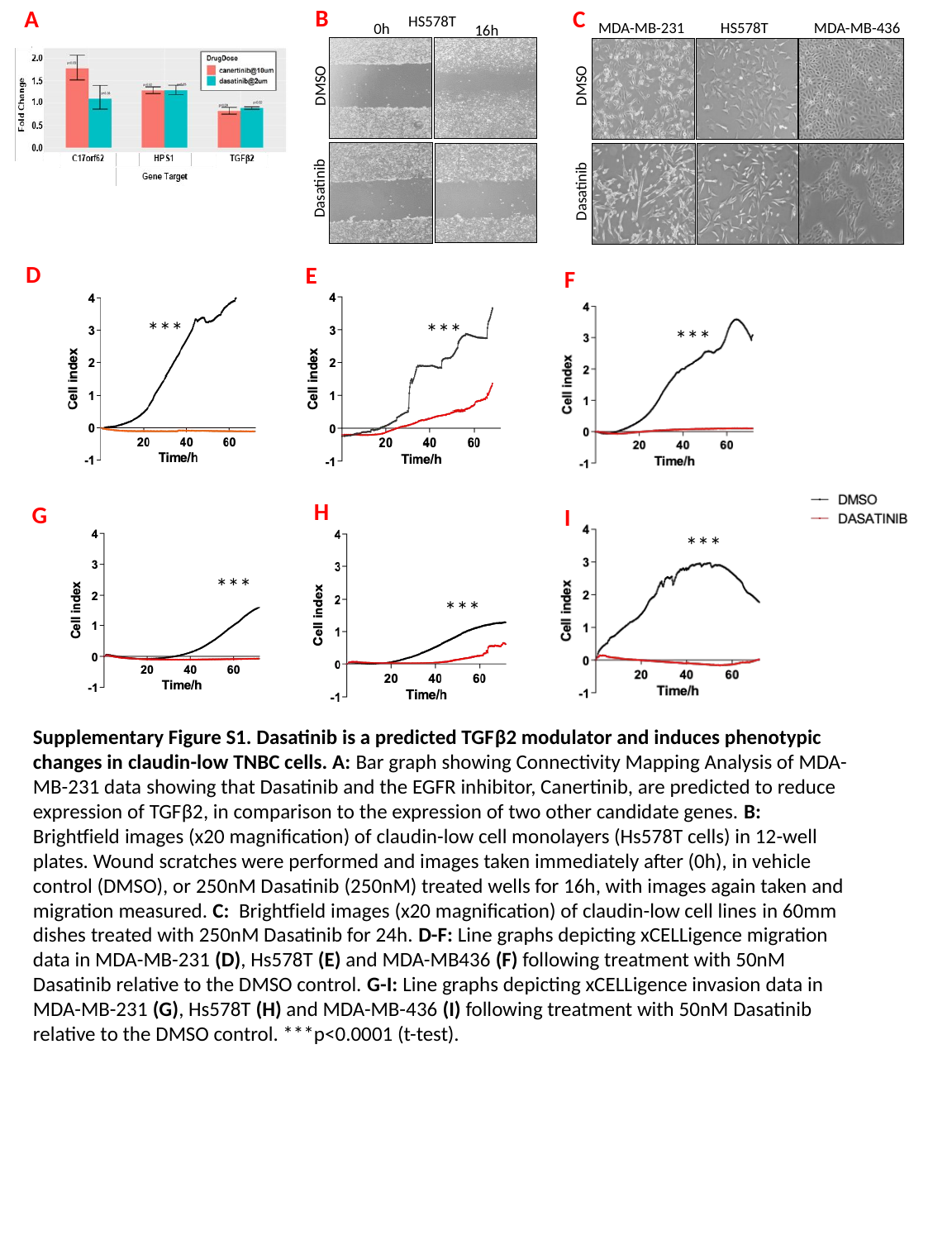

B
HS578T
0h
16h
DMSO
Dasatinib
A
C
MDA-MB-231
HS578T
MDA-MB-436
DMSO
Dasatinib
D
E
F
***
***
***
H
G
I
***
***
***
Supplementary Figure S1. Dasatinib is a predicted TGFβ2 modulator and induces phenotypic changes in claudin-low TNBC cells. A: Bar graph showing Connectivity Mapping Analysis of MDA-MB-231 data showing that Dasatinib and the EGFR inhibitor, Canertinib, are predicted to reduce expression of TGFβ2, in comparison to the expression of two other candidate genes. B: Brightfield images (x20 magnification) of claudin-low cell monolayers (Hs578T cells) in 12-well plates. Wound scratches were performed and images taken immediately after (0h), in vehicle control (DMSO), or 250nM Dasatinib (250nM) treated wells for 16h, with images again taken and migration measured. C:  Brightfield images (x20 magnification) of claudin-low cell lines in 60mm dishes treated with 250nM Dasatinib for 24h. D-F: Line graphs depicting xCELLigence migration data in MDA-MB-231 (D), Hs578T (E) and MDA-MB436 (F) following treatment with 50nM Dasatinib relative to the DMSO control. G-I: Line graphs depicting xCELLigence invasion data in MDA-MB-231 (G), Hs578T (H) and MDA-MB-436 (I) following treatment with 50nM Dasatinib relative to the DMSO control. ***p<0.0001 (t-test).
