# Supplementary material for: A SRC-slug-TGFβ2 signaling axis drives poor outcomes in triple-negative breast cancers
Source: Cell Commun Signal. 2024 Sep 26;22:454. doi: 10.1186/s12964-024-01793-6 (PMC11426005; doi:10.1186/s12964-024-01793-6)
Supplement: Supplementary file 5 — Supplementary Material 5 [file 12964_2024_1793_MOESM5_ESM.pptx]

## Slide 1
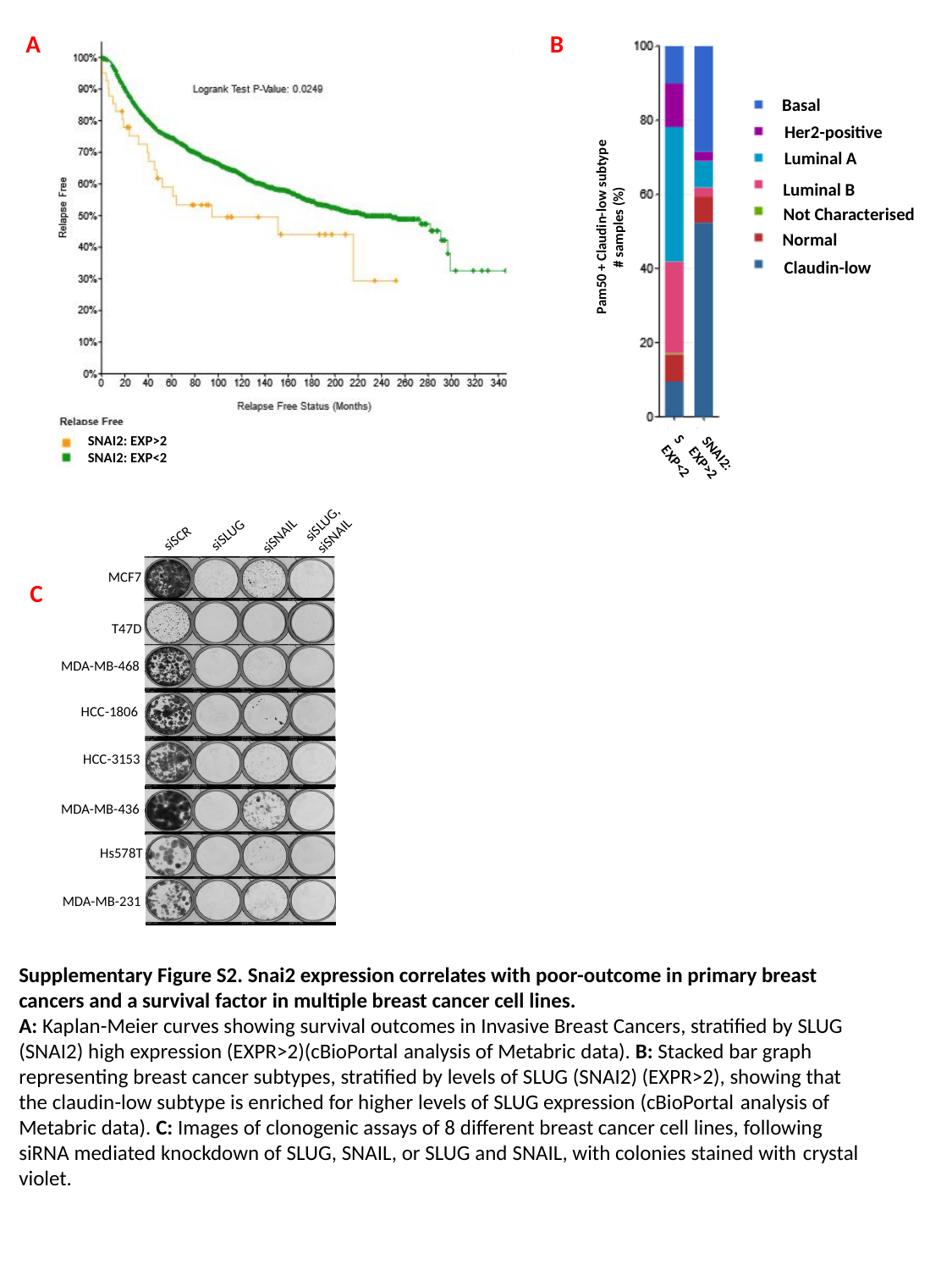

B
A
Basal
Her2-positive
Luminal A
Luminal B
Not Characterised
Normal
Claudin-low
Pam50 + Claudin-low subtype
# samples (%)
SNAI2: EXP>2
SNAI2: EXP<2
SNAI2: EXP<2
SNAI2: EXP>2
siSLUG, siSNAIL
siSLUG
siSNAIL
siSCR
MCF7
T47D
MDA-MB-468
HCC-1806
HCC-3153
MDA-MB-436
Hs578T
MDA-MB-231
C
Supplementary Figure S2. Snai2 expression correlates with poor-outcome in primary breast cancers and a survival factor in multiple breast cancer cell lines.
A: Kaplan-Meier curves showing survival outcomes in Invasive Breast Cancers, stratified by SLUG (SNAI2) high expression (EXPR>2)(cBioPortal analysis of Metabric data). B: Stacked bar graph representing breast cancer subtypes, stratified by levels of SLUG (SNAI2) (EXPR>2), showing that the claudin-low subtype is enriched for higher levels of SLUG expression (cBioPortal analysis of Metabric data). C: Images of clonogenic assays of 8 different breast cancer cell lines, following siRNA mediated knockdown of SLUG, SNAIL, or SLUG and SNAIL, with colonies stained with crystal violet.
