# Supplementary material for: A SRC-slug-TGFβ2 signaling axis drives poor outcomes in triple-negative breast cancers
Source: Cell Commun Signal. 2024 Sep 26;22:454. doi: 10.1186/s12964-024-01793-6 (PMC11426005; doi:10.1186/s12964-024-01793-6)
Supplement: Supplementary file 6 — Supplementary Material 6 [file 12964_2024_1793_MOESM6_ESM.pptx]

## Slide 1
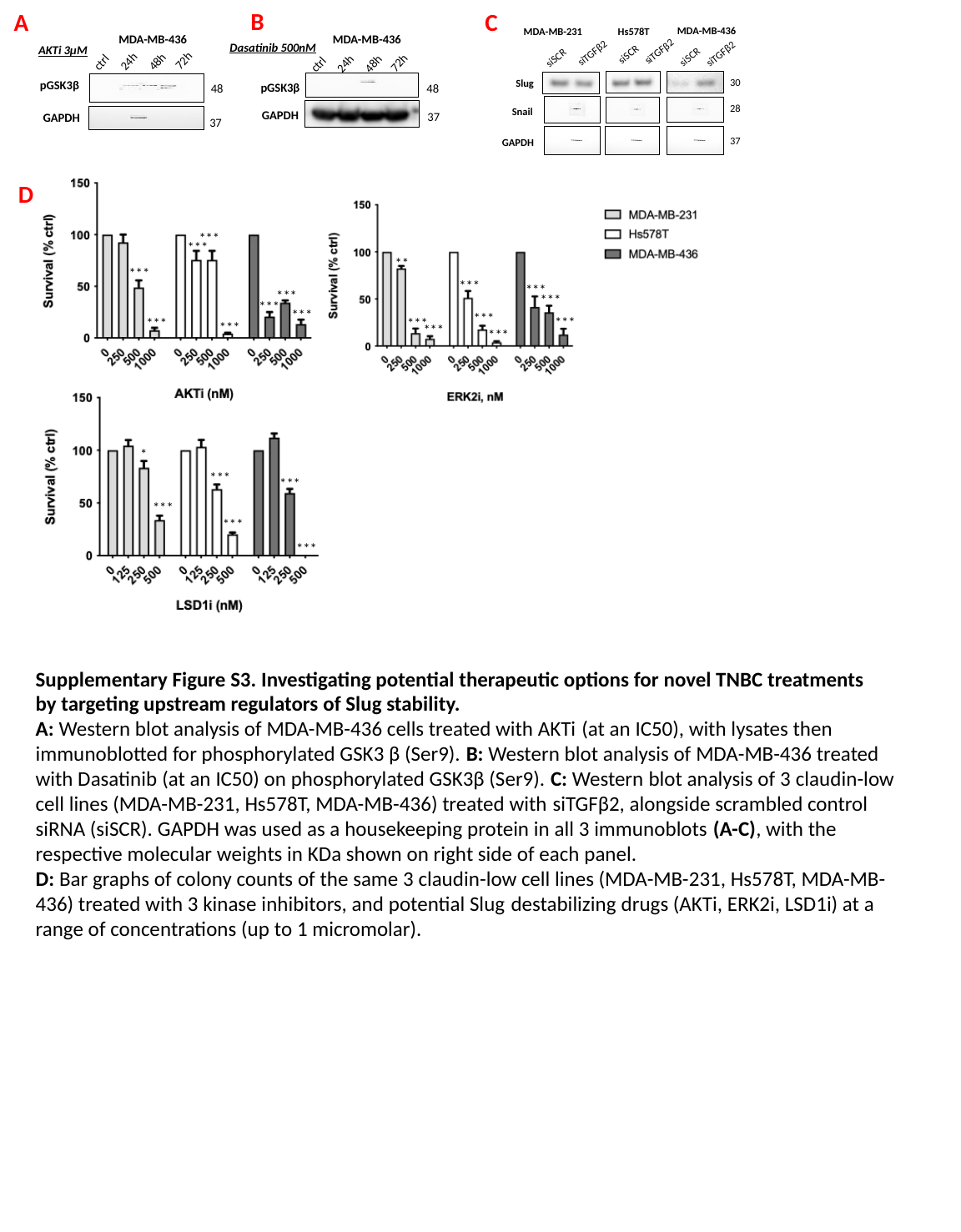

B
C
A
MDA-MB-436
MDA-MB-231
Hs578T
siTGFβ2
siTGFβ2
siTGFβ2
siSCR
siSCR
siSCR
Slug
Snail
GAPDH
30
28
37
MDA-MB-436
AKTi 3µM
72h
24h
48h
ctrl
pGSK3β
48
GAPDH
37
MDA-MB-436
Dasatinib 500nM
72h
24h
48h
ctrl
pGSK3β
48
GAPDH
37
D
***
***
**
***
***
***
***
***
***
***
***
***
***
***
***
***
***
*
***
***
***
***
***
Supplementary Figure S3. Investigating potential therapeutic options for novel TNBC treatments by targeting upstream regulators of Slug stability.
A: Western blot analysis of MDA-MB-436 cells treated with AKTi (at an IC50), with lysates then immunoblotted for phosphorylated GSK3 β (Ser9). B: Western blot analysis of MDA-MB-436 treated with Dasatinib (at an IC50) on phosphorylated GSK3β (Ser9). C: Western blot analysis of 3 claudin-low cell lines (MDA-MB-231, Hs578T, MDA-MB-436) treated with siTGFβ2, alongside scrambled control siRNA (siSCR). GAPDH was used as a housekeeping protein in all 3 immunoblots (A-C), with the respective molecular weights in KDa shown on right side of each panel.
D: Bar graphs of colony counts of the same 3 claudin-low cell lines (MDA-MB-231, Hs578T, MDA-MB-436) treated with 3 kinase inhibitors, and potential Slug destabilizing drugs (AKTi, ERK2i, LSD1i) at a range of concentrations (up to 1 micromolar).
