# Supplementary material for: A SRC-slug-TGFβ2 signaling axis drives poor outcomes in triple-negative breast cancers
Source: Cell Commun Signal. 2024 Sep 26;22:454. doi: 10.1186/s12964-024-01793-6 (PMC11426005; doi:10.1186/s12964-024-01793-6)
Supplement: Supplementary file 7 — Supplementary Material 7 [file 12964_2024_1793_MOESM7_ESM.pptx]

## Slide 1
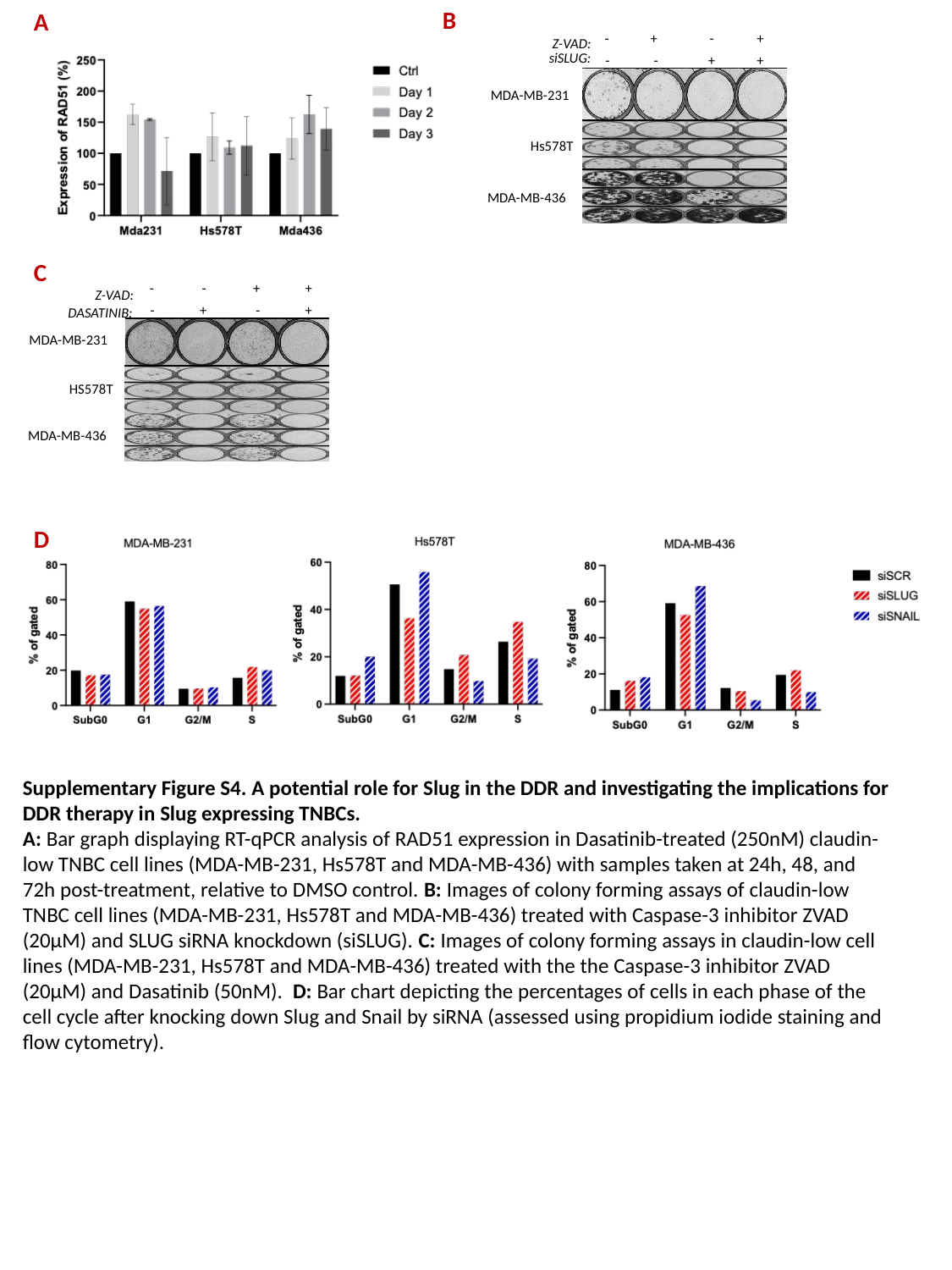

B
A
-
+
-
+
Z-VAD:
siSLUG:
+
-
-
+
MDA-MB-231
Hs578T
MDA-MB-436
C
-
-
+
+
Z-VAD:
-
+
-
+
DASATINIB:
MDA-MB-231
HS578T
MDA-MB-436
D
Supplementary Figure S4. A potential role for Slug in the DDR and investigating the implications for DDR therapy in Slug expressing TNBCs.
A: Bar graph displaying RT-qPCR analysis of RAD51 expression in Dasatinib-treated (250nM) claudin-low TNBC cell lines (MDA-MB-231, Hs578T and MDA-MB-436) with samples taken at 24h, 48, and 72h post-treatment, relative to DMSO control. B: Images of colony forming assays of claudin-low TNBC cell lines (MDA-MB-231, Hs578T and MDA-MB-436) treated with Caspase-3 inhibitor ZVAD (20μM) and SLUG siRNA knockdown (siSLUG). C: Images of colony forming assays in claudin-low cell lines (MDA-MB-231, Hs578T and MDA-MB-436) treated with the the Caspase-3 inhibitor ZVAD (20μM) and Dasatinib (50nM).  D: Bar chart depicting the percentages of cells in each phase of the cell cycle after knocking down Slug and Snail by siRNA (assessed using propidium iodide staining and flow cytometry).
