# Supplementary material for: A SRC-slug-TGFβ2 signaling axis drives poor outcomes in triple-negative breast cancers
Source: Cell Commun Signal. 2024 Sep 26;22:454. doi: 10.1186/s12964-024-01793-6 (PMC11426005; doi:10.1186/s12964-024-01793-6)
Supplement: Supplementary file 8 — Supplementary Material 8 [file 12964_2024_1793_MOESM8_ESM.pptx]

## Slide 1
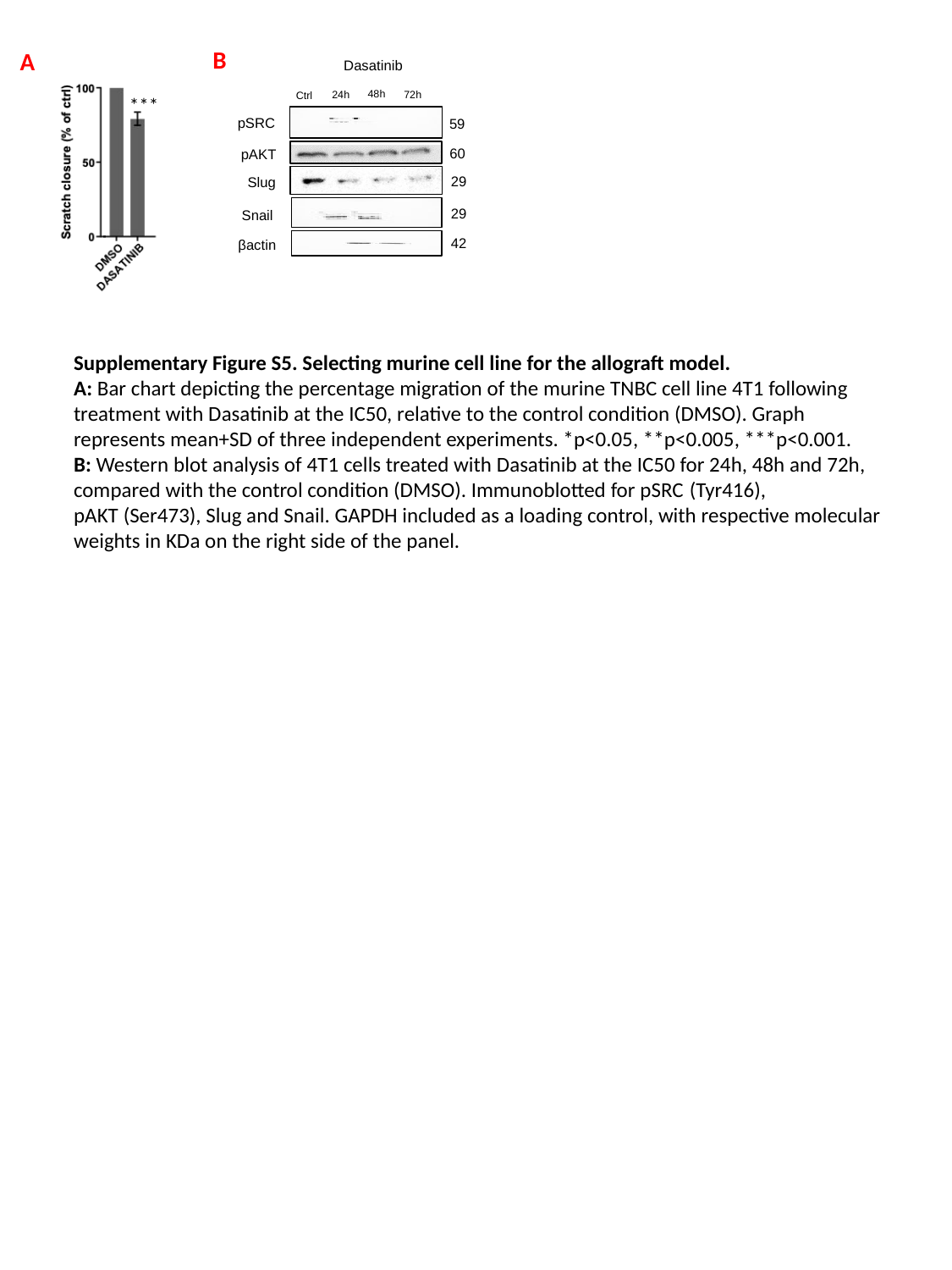

B
A
Dasatinib
pSRC
59
60
pAKT
29
Slug
29
Snail
42
βactin
48h
72h
24h
Ctrl
***
Supplementary Figure S5. Selecting murine cell line for the allograft model.
A: Bar chart depicting the percentage migration of the murine TNBC cell line 4T1 following treatment with Dasatinib at the IC50, relative to the control condition (DMSO). Graph represents mean+SD of three independent experiments. *p<0.05, **p<0.005, ***p<0.001.
B: Western blot analysis of 4T1 cells treated with Dasatinib at the IC50 for 24h, 48h and 72h, compared with the control condition (DMSO). Immunoblotted for pSRC (Tyr416), pAKT (Ser473), Slug and Snail. GAPDH included as a loading control, with respective molecular weights in KDa on the right side of the panel.
